# Development of a Drug-Response Modeling Framework to Identify Cell Line Derived Translational Biomarkers That Can Predict Treatment Outcome to Erlotinib or Sorafenib

**DOI:** 10.1371/journal.pone.0130700

**Published:** 2015-06-24

**Authors:** Bin Li, Hyunjin Shin, Georgy Gulbekyan, Olga Pustovalova, Yuri Nikolsky, Andrew Hope, Marina Bessarabova, Matthew Schu, Elona Kolpakova-Hart, David Merberg, Andrew Dorner, William L. Trepicchio

**Affiliations:** 1 Department of Translational Medicine, Takeda Pharmaceuticals International Co., 35 Landsdowne St., Cambridge, Massachusetts, 02139, United States of America; 2 Thomson Reuters, 777 E. Eisenhower Parkway, Ann Arbor, Michigan, 48108, United States of America; University of Queensland Diamantina Institute, AUSTRALIA

## Abstract

Development of drug responsive biomarkers from pre-clinical data is a critical step in drug discovery, as it enables patient stratification in clinical trial design. Such translational biomarkers can be validated in early clinical trial phases and utilized as a patient inclusion parameter in later stage trials. Here we present a study on building accurate and selective drug sensitivity models for Erlotinib or Sorafenib from pre-clinical in vitro data, followed by validation of individual models on corresponding treatment arms from patient data generated in the BATTLE clinical trial. A Partial Least Squares Regression (PLSR) based modeling framework was designed and implemented, using a special splitting strategy and canonical pathways to capture robust information for model building. Erlotinib and Sorafenib predictive models could be used to identify a sub-group of patients that respond better to the corresponding treatment, and these models are specific to the corresponding drugs. The model derived signature genes reflect each drug’s known mechanism of action. Also, the models predict each drug’s potential cancer indications consistent with clinical trial results from a selection of globally normalized GEO expression datasets.

## Introduction

It is well known that many cancer drugs are effective only in a subset of cancer patients, and selection of drug responsive biomarkers is crucial to find the right patients for the right drug. Also, early identification of biomarkers that can select sensitive and resistant patient subpopulations is particularly important in order to be able to test biomarker hypotheses in early clinical drug development and utilize those biomarkers during later clinical development to stratify patients into appropriate treatment arms. This will facilitate the development of companion diagnostic tests along with novel treatments. Therefore, it is ideal to discover drug response biomarkers prior to clinical trials using pre-clinical data sets, for example, by in vitro cell line screening of drug sensitivity.

Numerous studies of in vitro drug sensitivity screens [1–3] coupled with genomic/genetic profiling data have been conducted on the NCI60 cell line panel [4–11]. As the NCI-60 panel was of a limited size and tumor-type variety, more comprehensive and diverse cell line panels have been developed. Recently, two very large cell line panel studies were reported with several hundred cancer cell lines tested on dozens of oncology drugs [12, 13]. Powered by the comprehensive molecular characterization of the cancer cell lines in these panels and the drugs’ known mechanisms of action; both studies identified important candidate biomarkers for drug sensitivity [12, 13]. However, these studies did not directly generate drug sensitivity predictive models, nor did they validate the biomarkers on independent data sets obtained from treated patients where outcome of treatment was known.

Although there are a few successful examples of predictive gene expression signatures to predict disease prognosis, for example the 70-gene MammaPrint signature [14] and the 16-gene OncotypeDX test [15] for breast cancer, few highly reproducible gene expression signatures to select patients for appropriate drug treatment have been translated into useful clinical tests. This can be attributed to a wide number of factors, such as disease heterogeneity, robustness of selected features, and dealing with different molecular profiling platforms. Specially, inappropriate selection of modeling approaches employed during the discovery phase may also play a role. A recent FDA-led initiative [16] evaluated various gene expression modeling methods for predicting clinical endpoints (MAQCII: MicroArray Quality Contol II). In the project, 36 independent teams analyzed six microarray data sets to generate predictive models for classifying a sample with one of 13 endpoints. Using independent testing data, the study found that the biology of the end-point is the main performance-associated factor. Thus, 36 independent teams made poor predictions on complex end-points such as overall cancer survival and chemically-induced carcinogenesis [16].

The poor model performance could be improved if more appropriate modeling approaches for the complex clinical endpoints of interest were used. For instance, poor prediction of overall survival for Multiple Myeloma patients in the MAQCII study could be partly due to applying an arbitrary survival cutoff (24 month) for patients [16]. Both gene expression and overall survival in the Multiple Myeloma case are continuous variables, therefore one can build a regression-based prediction model. In fact, when an univariate Cox regression approach is used, it identifies a gene expression signature that significantly predicts a “high-risk” subgroup of patients [17]. This signature was later validated on several independent studies and on different regression-based approaches [18–21], highlighting the advantage of a regression approach without predefined class memberships.

In order to generate translational drug-sensitive models, e.g. building predictive models using cancer cell line data that can be used to predict patient response in clinical trials, more detailed considerations are needed along the model building/testing process. For example, as observed from the MAQC II study, a simple application of t-test for feature selection is not sufficient [16]. Instead, a resampling approach to gain robustness on feature selection is appropriate. Another frequent topic to address is that to build/test translational drug-sensitivity signatures, one often needs to normalize datasets generated from different profiling platforms. There are some well-accepted normalization approaches being developed [22, 23], and one needs to select the right method based on the individual projects with special considerations of the datasets.

In the current work, a combination of novel statistical and functional feature selection approaches were used to build in vitro gene transcription based predictive models, for Erlotinib and Sorafenib response using a 240 cancer cell line panel (OncoPanel: http://www.Eurofinspanlabs.com). IC50 values were used as dependent variables and gene expression data from untreated cells was used as independent variables to build the predictive models. A Partial Least Squares Regression (PLSR) modeling approach was utilized because it can effectively handle a high number of independent variables with minimal demands on sample size [24–26]. Importantly, a special splitting strategy was implemented to capture consensus features in the training dataset, followed by a pathway-based filtering step to highly reduce the signature gene set without losing model performance. In the present study, performance of the functional signature was validated to predict Erlotinib or Sorafenib patients’ response and linked it to progression free survival (PFS) in lung cancer patients from the BATTLE clinical trial [27]. The in vitro-derived predictive models demonstrated significant in vivo accuracy, were mechanistically linked to the drugs’ mechanism of action and were highly drug-specific.

## Results

### A PLSR-based modeling framework to build predictive models for drug sensitivities

The main goal of this study was to test whether drug sensitivity models derived from cell line data could be used to predict patient response to the drug. The whole cell line panel was used as the training dataset, and evaluation of the model performance was carried out using gene expression data generated from tumor samples of patients treated with the same drug. No information from the testing dataset was used in training the drug sensitivity predictive models.

Erlotinib was selected as a case study target as it has clear mechanism of action as an EGFR inhibitor, and because molecular data sets coupled with patient response to treatment are publicly available (the MD Anderson BATTLE clinic trial). A 240 tumor cell line panel (Oncopanel) was used to identify cell lines that were sensitive or resistant to Erlotinib treatment (using the median IC50 value as a cutoff if the IC50 distribution is normal-like, or using a data driven cutoff value if the IC50 distribution follows a bi-model distribution). The Oncopanel contains cancer cell lines covering a variety of cancer indications (S1 Fig). For each drug against each cell line in the panel, a cell proliferation assay was conducted using 10 doses of Erlotinib (3-fold dilution) and IC50 values were generated. For model building on drug sensitivity, baseline gene expression was used as the independent variable and IC50 as the dependent variable.

**Fig 1:**
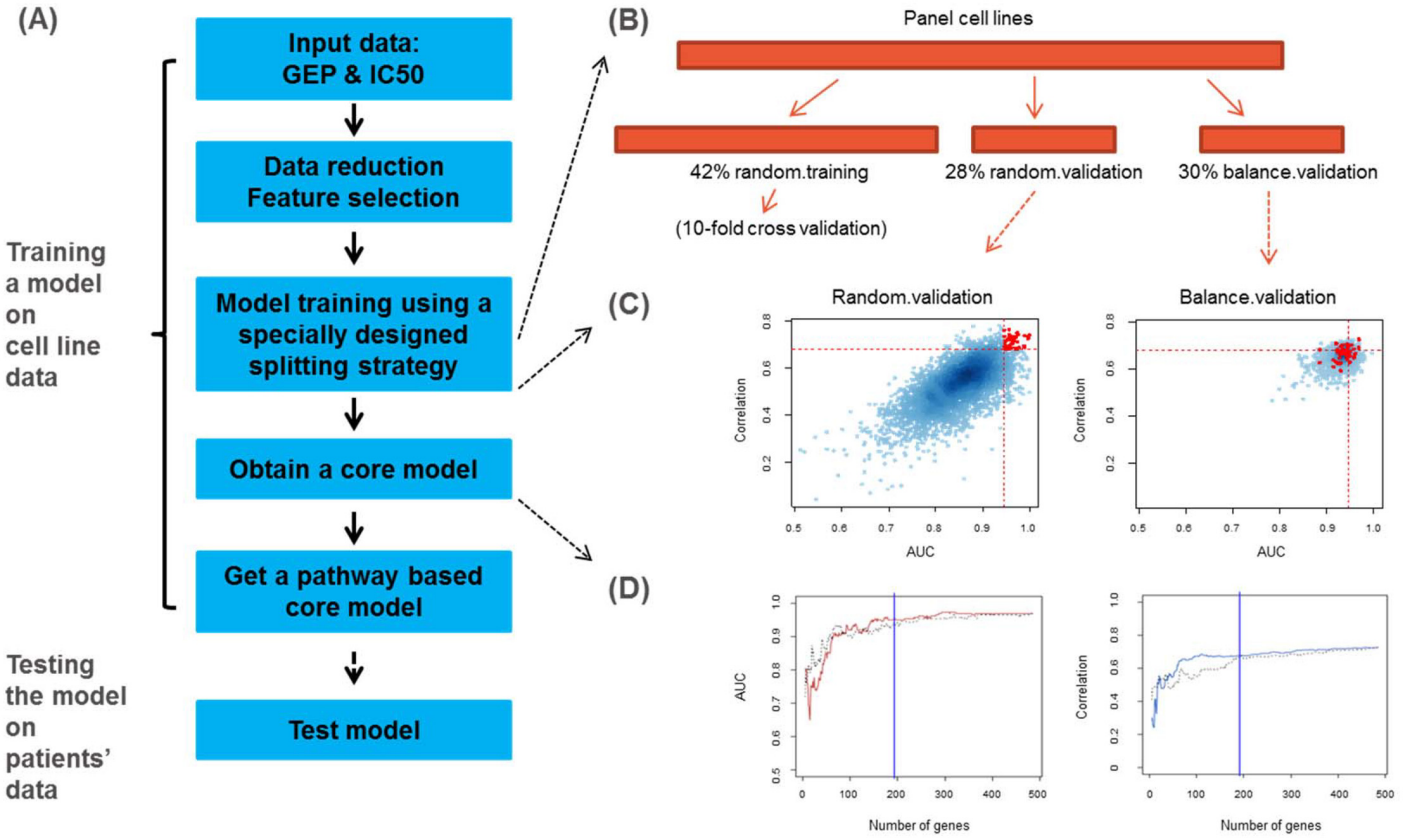
PLSR modeling workflow applied on 183 cancer cell lines on OncoPanel. (A). Flow chart on the model building and testing steps. (B). A specially designed splitting strategy divides the training dataset into random training, random validation and balance validation subsets. (C). Representative example of random validation and balance validation. Red points were top performing models on 1000 random splits on this balanced split, based on both AUC and correlation measures. (D). AUC and correlation cutoff selection for the core PLSR model.

A PLSR model workflow was developed and an Erlotinib sensitivity model was trained using OncoPanel data (Fig 1). The key steps shown on the left include multiple steps of data reduction, feature selection, a special splitting strategy to capture consistent features across the dataset, selection of least-overlapping top models, calculation of consensus genes weights followed by selection of the core signature gene set, and ontology enrichment filtering to obtain the pathway-based PLSR model (Fig 1A).

#### Data reduction

First, an RMA normalization of the baseline gene expression of the OncoPanel cell lines was conducted. Next, an intensity cutoff of 40% of the whole genome to remove genes with low intensities was applied. Third, a variance cutoff of 1 to keep genes with the highest variability of expression among the panel cell lines was applied. This data reduction step narrowed down the number of probesets by an order of magnitude from 54,675 to 3,787.

#### Feature selection

Feature selection was conducted on the 3787 probesets based on correlation between gene expression level and drug response (log_2_(IC50)) for all cell lines. For each probeset, permutation testing was run by randomly assigning a response sign to the cell lines panel. After Fisher's transformation (f = 0.5 * ln ((1+r) / (1-r), where r is the correlation coefficient on each permutation), the permutation resulted in f-values that follow a normal distribution and a raw p-value of each gene was calculated based on the fitted mean and standard deviation from the permutation data. An adjusted p-value was calculated using Benjamini-Hochberg control of false discovery rate [28]. Feature probesets had to pass a raw p-value cutoff of 0.01. Then, the highest intensity probesets were selected as representative for each gene; typically these were also the highest variance probesets for the gene. As a result, 485 genes were selected and used as the input gene set for PLSR model training.

#### A splitting strategy to capture consensus features in the training dataset

In order to select the top-performing models that also capture consensus features in the dataset, a special splitting approach was developed. First, a “balanced split” was conducted to divide the data into training (70%) and balance validation (30%) subsets: we first equally divided the cell lines into three groups by IC50 values, then conducted random splits on each group to get training and validation subsets. When merging the training and validation subsets from these three groups, both training and validation subsets having some sensitive and some resistant cell lines (termed “balanced split”) were generated. The training subset was then further divided into random training (60% of training) and random validation (40% of the training) subsets. Overall, the training set was divided into three parts: random training (42%), random validation (28%) and balance validation (30%) subsets (Fig 1B). Hundreds of thousands of splits were generated, then the top models were selected by comparing model performance on both random validation and balance validation results.

#### Identifying the not-significant-overlapping models

The best models were selected based on multiple parameters, using both random validation and balance validation results (Fig 1C). The selection criteria were as follows: 1. the models should have good performance on random validation on both correlation and Area Under Curve (AUC) measures (red spots in Fig 1C). The correlation is defined as *Pearson* correlation between experimental and predicted IC50s. The AUC measure uses a data-driven cutoff to divide cell lines into sensitive- and resistant-groups using experimental IC50s, and use a series of cutoffs on predicted IC50s to define predicted sensitive- and resistant groups, to generate the Receiver Operating Characteristic (ROC) curve and calculate AUC values. In this sense, although a regression-based modeling framework was built, some classification measures to help select top models were still used; 2. Among all splits, a correlated AUC and correlation measures on random validation (Fig 1C-left panel) were preferred; 3. Balance validation (from balanced split) should have much more narrow performance distribution than the random validation (Fig 1C-right panel); 4. The model performance in balance validation (Fig 1C-right panel) should not be inferior to random validation (Fig 1C-left panel), which may indicate over-fitting on the random training and random validation; 5. The models should have relatively high performance among all splits in balance validation, when comparing the distribution on Fig 1C right versus Fig 1C left panels.

Five top balance split resulted model sets (each contains 1000 random splits) were selected based on all five criteria out of 150 initial balance split model sets. Then, one top model with best correlation between predicted and experimental IC50 in balance validation set was selected as a top model in each of the top five model sets, resulting in 5 top models from 150*1000 = 150000 total splits. Pairwise overlapping scores among these models were then calculated (the overlapping score was defined as Jaccard similarity index), on the real random training cell line sets as well as generated an overlapping score distribution on randomly select two sets with the same size of cell lines as in the random training (a permutation test). In order to remove top models that were significantly overlapping to each other, a 90% quantile cutoff on pairwise overlapping scores was used as a significant overlapping cutoff.

#### Calculating consensus genes weights and selecting the core signature gene set

There are multiple potential ways to identify key biomarker genes from a single training/testing dataset, for example, several sparse PLSR approaches were developed by Chun and Keles [29, 30], Lê Cao et al.[31, 32], and Witten and co-workers [33], which can effectively perform internal variable selections. On the other hand, since a key goal in the current work is to capture consensus information among a pan-cancer panel of cell lines, we chose to use a forward selection procedure to identify consistent gene weights among not-significantly overlapping top models. After ranking models by the split strategy described above, genes’ consensus weights were calculated using the Singular Value Decomposition method (SVD) [34] to summarize genes’ loading values from each top model. Then, genes’ loading values from each top model were compared against genes’ consensus weightings, as well as all pairwise comparisons of genes’ loadings between each pair of top models (S2 Fig). An individual top model that showed the highest similarity to the consensus weightings was selected as a representative model for later steps.

Starting from the five highest weighted genes and adding one gene at a time, the representative PLSR model was retrained/retested by increasing signature gene size one at a time (a forward selection approach). The core PLSR model was identified as an early plateau point on the model performance curves on both AUC and correlation measures (Fig 1D). The resulting core PLSR model contained 191 genes.

#### Generation of a pathway-based “functional” PLSR model

A subset of the core PLSR model as the pathway-based PLSR model was selected using the functional analysis platform MetaCore (Thomson Reuters) [35]. The 191 genes of the core model were subjected to enrichment analysis in the canonical pathway maps ontology. The 51 genes situated on statistically significant (p-value <0.01) pathway maps were selected as the pathway-based classifier and retested on the same OncoPanel cell lines. Importantly, the EGFR ligand (NRG1) was among the signature genes, which is consistent with the fact that Erlotinib is an anti-EGFR compound.

**Fig 2:**
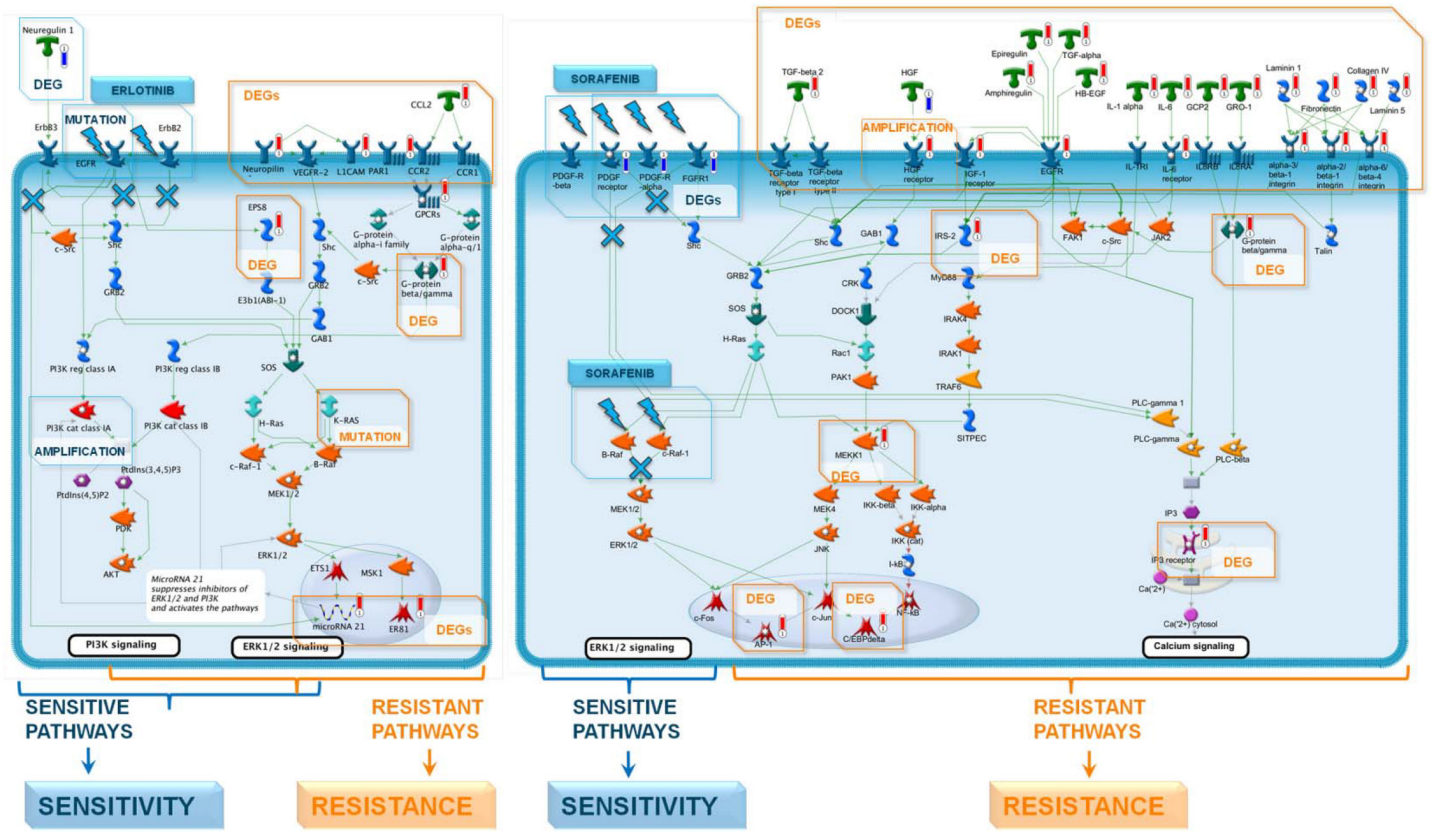
Causal network to depict functional relations between sensitivity-specific and resistance-specific signature genes. The network was reconstructed from canonical signaling pathways regulated by signature genes and a signature specific direct interaction network. Sensitivity-specific signature genes are highlighted with blue thermometers, resistance-specific genes with red thermometers.

In the 51-gene signature (S1 Table), 22 genes correlated positively and 29 genes negatively with IC50 data. Further the genes were referred to as resistance-specific and sensitivity-specific, correspondingly. In order to understand how the Erlotinib signature genes are related to the biology of drug mechanism of action, we connected the corresponding functional signatures genes into molecular networks, using data on canonical pathways and interactions from MetaCore (Thomson Reuters) as network building blocks (Fig 2; please see S3 Fig and S1 Text for details). The networks contained genes, whose variants are significantly associated with the corresponding drug response. Specifically, EGFR mutations and amplifications of PI3KA class A are associated with sensitivity and K-Ras mutations with resistance to Erlotinib (Fig 2A, please, see S3 Table and S2 Text for details). This is consistent with the results from the BATTLE study, where EGFR mutations were associated with better response to Erlotinib [27].

### Building a Sorafenib sensitivity model

To test for drug-specificity of the Erlotinib PLSR model, a predictive model to Sorafenib was built using data from Oncopanel cell lines treated with Sorafenib to generate IC50 values as well as the same baseline gene expression data and modeling framework as for Erlotinib. Therefore, the data reduction is the same for both Erlotinib and Sorafenib and the models are directly comparable.

Unlike Erlotinib’s IC50 distribution (S5 Fig(A)), the Sorafenib IC50 values follow a normal-like distribution (S5 Fig(B), upper panel). To better separate Sorafenib sensitive vs resistant cases, the middle one-third of the cell line panel was removed before the model training process (S5 Fig(B), lower panel). Even with this filtering, it was still more difficult to identify a good predictive Sorafenib model compared to Erlotinib. An example of the random validation and balance validation performance of the Sorafenib model (S6 Fig) demonstrates it does not perform as well as the Erlotinib models (Fig 1C). For Sorafenib predictive model building, 903 feature genes were identified, with 550 core model genes, and eventually 113 genes for the final pathway signature (S2 Table).

A network of Sorafenib signature genes was generated using protein-protein interactions and canonical pathway information (Fig 2B, please see S1 Text and S4 Fig for details). The networks contained genes with variants significantly associated with the corresponding drug response. The genes positively correlated with IC50s and associated with resistance, tend to populate signaling pathways parallel or cross-talking to the drug target signaling pathway (Fig 2). Activation of parallel pathways by overexpression or mutations conveys the common resistance mechanism for Sorafenib. One of the Sorafenib-resistance pathways, EGFR signaling, is represented by 4 overexpressed ligands. Moreover, different genetic events in EGFR signaling are associated with Sorafenib resistance among OncoPanel cell lines (S3 Table and S2 Text for details). Importantly, EGFR mutations were associated with worse response to Sorafenib in the BATTLE study.

### Testing model performance of Erlotinib and Sorafenib predictive models using the BATTLE clinical trial as an independent testing dataset

The BATTLE clinical trial data was used as an independent testing dataset, to evaluate the performance of the OncoPanel cell line data derived drug sensitivity models. In this Phase II clinical trial [27], subsets of 255 NSCLC patients were treated with either Erlotinib, Vandetanib, Sorafenib or Erlotinib + bexaroten combination. Among the 255 patients, there were 131 patients with tumor samples sufficient for molecular profiling and clinically evaluable (GSE33072). Among them, data from 25 patients in the Erlotinib arm and 39 patients in the Sorafenib arm was usable for PLSR model testing.

The baseline tumor gene expression data in patients was generated using the Affymetrix HG Gene 1.0 ST array, which is a different platform than the U133plus2 array used in the OncoPanel experiments. To address the issue of platform incompatibility, the Affymetrix “U133PlusVsHuGene_BestMatch” file was used to identify matching probesets between U133plus2 and HG Gene 1.0 ST arrays, then a quantile normalization of the BATTLE data against OncoPanel gene expression was performed. All 51 genes from the Erlotinib functional pathway-based model were present in the BATTLE dataset, as well as all 113 Sorafenib signature genes.

The cutoffs of Erlotinib or Sorafenib predicted scores were data-driven by re-predicting IC50s on the Oncopanel cell lines, using the corresponding drug sensitivity models (S7 Fig). The distribution of Sorafenib predicted IC values do not have a clear separation point, so a somewhat arbitrary cutoff of 2 was selected. This happens to be the same boundary value for experimental IC50s after removing the middle one-third cell lines for Sorafenib predictive model training, providing some supporting evidence on selecting this cutoff. Since the BATTLE patients’ baseline gene expression was normalized against the OncoPanel gene expression using the whole genome, the cutoffs defined from the OncoPanel dataset were applied to the BATTLE dataset.

**Fig 3:**
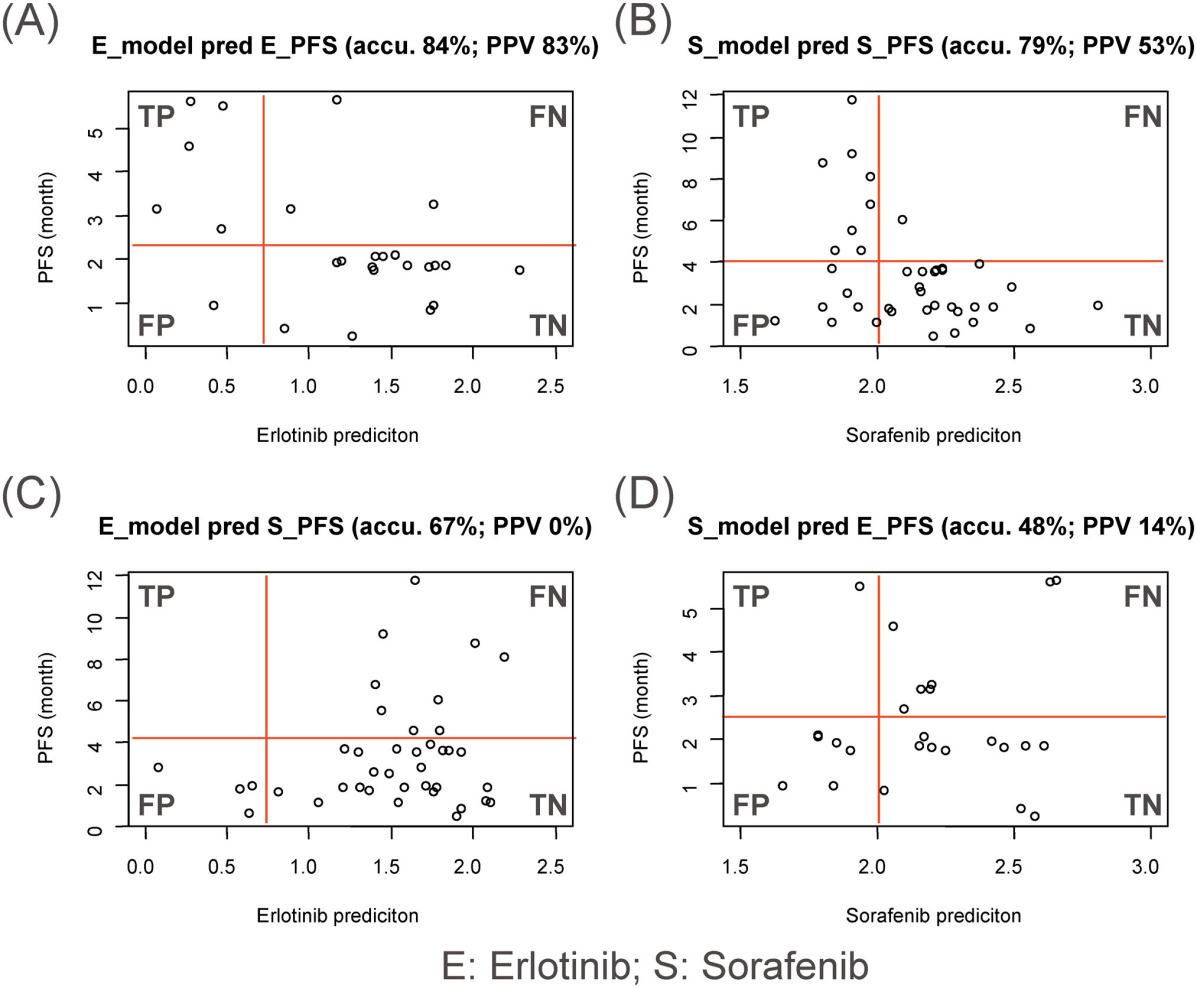
PLSR models performance in predicting Erlotinib-treated patient survival in the BATTLE trial. A. Erlotinib model predicting Erlotinib treated patients; B. Sorafenib model predicting Sorafenib treated patients; C. Erlotinib model predicting Sorafenib treated patients; and D. Sorafenib model predicting Erlotinib treated patients. TP: true positive; FP: false positive; TN: true negative; FN: false negative; PPV: positive predictive value.

The patients’ PFS cutoffs for Erlotinib and Sorafenib were selected at 2.4 and 4.2 months based on their PFS distributions, respectively (S8 Fig). We are aware that these cutoff value selections are somewhat arbitrary, since the Erlotinib or Sorafinib arm in the BATTLE clinical trials has small patient numbers (25 and 39 patients for Erlotinib and Sorafenib arms, respectively). However, as shown as red horizontal lines in S8 Fig and Fig 3, these cutoffs showed reasonable PFS separation on patients treated by Erlotinib or Sorafenib, respectively.

#### Erlotinib model predicts Erlotinib response

As shown in Fig 3A, the Erlotinib model built from the OncoPanel Erlotinib screen data (IC50s) predicted response for the Erlotinib treated patients reasonably well. The Erlotinib model performance for accuracy, sensitivity, specificity, positive predictive value, and negative predictive value are 84%, 63%, 94%, 83% and 84%, respectively.

#### Sorafenib model predicts Sorafinib response

Similarly, the Sorafenib model built from OncoPanel Sorafenib screen data (IC50s) can predict drug response for the 39 Sorafenib treated patients (Fig 3B). The Sorafenib model performance for accuracy, sensitivity, specificity, positive predictive value, and negative predictive value are 79%, 89%, 77%, 53% and 96%, respectively.

#### Erlotinib model does not predict Sorafenib response

The Erlotinib model was further tested to predict the response of Sorafenib treated patients. For these heavily pre-treated patients in the BATTLE clinical trial, both the PFS and model-predicted scores suggested that the majority of the patients are not Erlotinib sensitive. The overall accuracy of the Erlotinb model in predicting Sorafenib treatment outcome was 64% (Fig 3C). Interestingly, the Erlotinib model predicted four patients in the Sorafenib treated arm to be Erlotinib sensitive but none of them was actually sensitive to Sorafenib treatment, which corresponds to a positive predictive value of 0%. In comparison, when the Erlotinib model was used to predict Erlotinib treated patients’ response, the positive predictive value was 83%.

#### Sorafenib model does not predict Erlotinib response

The Sorafenib model failed to predict the BATTLE Erlotinib treated patients’ response, with a positive predictive value of 14% and an overall accuracy of 48% (Fig 3D). It is worth noting that even in the case of using the Sorafenib model to predict Sorafenib treated patients’ response, the positive predictive value was only 53% (much lower than that of the Erlotinb model’s 83%). On the other hand, the Sorafenib’s model had a very high negative predictive value at 96%, so that the overall model accuracy was 79% (still slightly lower than Erlotinib model’s 84%).

Putting together, the Erlotinib or Sorafenib models trained from Oncopanel data can be used to predict patients’ response upon corresponding treatments. However, the models performed poorly in cross-evaluation (i.e. Sorafenib signature for predicting Erlotinib response and vice versa). This suggests that both the Erlotinib and the Sorafenib models are drug specific.

### Using Erlotinib and Sorafenib drug sensitivity models to predict progression free survival (PFS) in the BATTLE clinical trial

Another way to assess cell line data derived drug sensitivity models was to test the models for their ability to predict PFS. Patients in the BATTLE clinical trial were assigned to a marker positive (drug sensitive) or a marker negative (drug resistant) sub-group based on the corresponding PLSR drug predicted sensitivity scores, then marker +/- patient groups were compared based on the clinical output (PFS).

**Fig 4:**
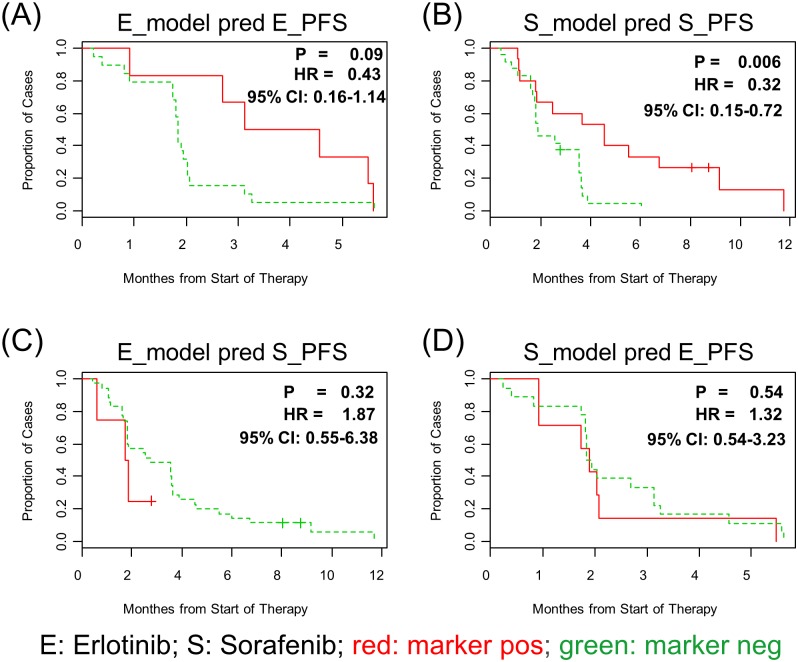
Survival analysis on biomarker identified treatment sensitive/resistant sub-groups. A. Using the Erlotinib model to stratify Erlotinib treated patients; B. Using Sorafenib model to stratify Sorafenib treated patients; C. Using Erlotinib model to stratify Sorafenib treated patients; and D. Using Sorafenib model to stratify Erlotinib treated patients.

When the Erlotinib model is used to stratify BATTLE patients in the Erlotinib arms, the median PFS for the model-predicted Erlotinib-sensitive patient group was 3.84 month while the PFS for model-predicted Erlotinib-resistant patients was 1.84 month, corresponding to a p-value of 0.09 and Hazard ratio of 0.43 (95% CI, 0.16 to 1.14, Fig 4A). The median PFS for all the patients in the BATTLE trial was 1.90 month, suggesting that the Erlotinib model, indeed, selected the patient group with twice as long survival. However, the survival difference between model-predicted Erlotinib sensitive vs resistant groups is not statistical significant (p-value at 0.09 and 95% CI of Hazard ratio at 0.16 to 1.14). Since all the patients in the Erlotinb treatment arm were EGFR wild-type, it was not possible to assess the Erlotinib models ability to predict outcome relative to the EGFR mutation biomarker. On the other hand, the current gene expression based biomarker works reasonably well on separating Erltonib sensitive versus resistant patients.

When using the Sorafenib model to predict PFS of BATTLE patients in the Sorafenib arm, the model identified marker-sensitive group had a survival benefit of 2.66 months (PFS) over the marker-resistant group (Fig 4B), with a p-value of 0.006 and a Hazard-ratio of 0.32 (95% CI, 0.15 to 0.72). The median PFS survival was 4.53 and 1.87 months, for marker-sensitive and marker-resistant groups, respectively.

Importantly, the signatures were drug-specific and did not predict across arms. The Erlotinib predictive model failed to separate marker-sensitive vs. marker-resistant groups for Sorafenib treatment arm and vice versa, suggesting that the predictive models are drug specific (Fig 4C and 4D).

### Applying drug sensitive models to predict percentage of drug sensitive samples in different cancer indications

After the Erlotinib and Sorafenib sensitivity models were tested using the BATTLE clinical trial data, they were further used to predict patients’ tumor samples from gene expression data in the public domain. The percentage of predicted Erlotinib or Sorafenib sensitive samples were generated based on cancer indications, and a higher percentage of predicted drug sensitivity for a cancer type was considered as a potential indication that the drug may work well on treating that type of cancer.

An internal data repository was built based on the tranSMART translational medicine platform [36] using data from public GEO datasets. As GEO microarray datasets are not uniformly processed, various methods were proposed to remove batch effects among studies (please see Lazar’s recent review [22] for more information). We choose the fRMA approach [37] for globally normalizing GEO datasets for two reasons: (1). fRMA uses a “frozen” reference set that makes it very easy to incrementally normalize new datasets; (2). fRMA uses the same approach as in RMA that is used for single dataset normalization. After global normalization, samples in the GEO datasets can be merged into cancer indications (see Materials & Methods for details). In total, 484 GEO studies with 16096 samples were normalized on Affymetrix microarray platform U133plus2 and merged into various cancer indications.

Extensive manual curation and text mining were performed to standardize metadata on patient and clinic features. For the purpose of applying the drug sensitivity models on patients’ cancer samples, our quality control contains the following aspects: (1). Remove cell line data; (2). Remove normal tissue samples; (3). In some solid tumor studies, the samples were actually collected from blood or saliva—we also removed data from these studies. After these quality control steps, the resulting 11331 samples was categorized into various cancer indications.

Each sample’s baseline gene expression was used to predict its potential Erlotinib or Sorafenib response, and percentage of predicted drug sensitive samples were calculated upon each cancer indication. Overall, differential pattern of drug sensitive indications is generally consistent with clinical trials outcomes for Erlotinib and Sorafenib, respectively (Table 1). Lung cancer was predicted to have 15.81% of Erlotinib sensitive samples, which is consistent with the fact that Erlotinib was approved to treat lung cancer patients. On the other hand, kidney cancer was predicted to have 0.46% of Erlotinib sensitive samples, which is consistent with a recent Erlotinib Phase III clinical trial that showed single agent Erlotinib treatment failed to show benefits for kidney cancer patients. For Sorafenib, 31.76% and 24.77% of kidney and liver cancer samples were predicted to be Sorafenib sensitive, respectively (Table 1), which are consistent with FDA approval of Sorafenib on treating kidney and liver cancer patients.

**Table 1:** Predicted percentage of Erlotinib and Sorafenib sensitive samples for some cancer indications from Gene Expression Omnibus datasets.

Cancer Type	Number of samples	Pred. Erlotinib Sen. percentage	Pred. Sorafenib Sen. percentage
***FDA approved Erlotinib or Sorafenib indications*:**			
Lung Cancer	329	15.81	0.61
Liver Cancer	85	0.00	31.76
Kidney Cancer	218	0.46	24.77
***Additional indications*:**			
Head and Neck Cancer	168	94.05	12.50
Bladder Cancer	102	41.18	3.92
Acute lymphoblastic leukemia	516	4.26	64.73
Diffuse Large B-Cell Lymphoma	816	2.45	30.51
Acute myeloid leukemia	1118	0.45	15.74
Multiple myeloma	596	0	27.01
Bone Cancer	88	0.00	29.55
Breast Cancer	1668	5.64	6.47
Colorectal Cancer	948	0.11	0.21
Pancreatic Cancer	75	0.00	1.33

The predictive models were derived from cell line Oncopanel expression data. Patient data normalization is described in the result section.

Several interesting predictions on other cancer types were also observed. For example, 94.05% of head & neck samples were predicted to be Erlotinib sensitive. Interestingly, there are a number of ongoing Erlotinib trials for this cancer type (http://www.clinicaltrials.gov/). In addition, Sorafenib model predictions suggested there are a high percentage of drug sensitive samples in bone cancer and haematopoietic neoplasms (Table 1). There were 5.64% and 6.47% of breast cancer samples predicted to be sensitive to Erlotinib or Sarafenib treatment, respectively. Given the high total number of breast cancer patients, these sub-population of patients may still be clinical meaningful. On the other hand, colorectal or pancreatic cancer was predicted to have very low percentage of Erlotinib or Sorafenib sensitive cases (Table 1).

## Discussion

### Key features of the modeling framework reported in this work

#### Using cell line derived drug sensitivity models to predict patients’ response

Systematic differences between expression patterns in human tumors and in vitro tumor cell line models [38, 39] contributes to uncertainty of predictive performance of signatures generated from one source of samples (cell lines) and applied to another source (primary tumors). This issue was addressed in a number of previous cell line based signature generation studies. For example, in one NCI-60 study, the “co-expression extrapolation” (COXEN) algorithm was developed for selecting the few genes expressed in sync between cell lines and primary tumors [3]; both tumor and cell lines expression patterns were used for deriving a multi-gene predictor. One potential limitation for the COXEN algorithm was its feature selection approach: clustering analysis was conducted on both preclinical and clinical data to select input genes for model training [3]. In contrast, our model was built entirely on cell line panel data, so the BATTLE clinical dataset can be used as an independent testing dataset.

Recently, Blumenschein and coworkers identified a Sorafenib sensitivity signature from 68 NSCLC cell lines, then applied the signature to retrospectively stratify Sorafenib sensitive patients in the BATTLE clinical trial [40]. Our Sorafenib sensitivity model has similar performance in predicting patients’ response to Sorafenib. However, we built our model using a mixture of cancer cell types instead of NSCLC cell lines only. As a result, our Sorafenib model was further applied to predict potential Sorafenib sensitive cancer indications (Table 1), which was consistent with FDA approved Sorafenib indications.

#### A specially designed splitting strategy to help us identify consensus information for predictive model building

It was reasoned that if one can identify the consensus information within a training dataset, the resulting predictive model should be robust and have high predictive power. An important feature of our approach is a specially designed splitting strategy (division on random training, random validation and balance validation sample subsets), which creates balance validation subsets as well as random validation subsets. The feature selection was done on the whole dataset, so that the same feature genes could be used to train and compare models among different splits. This also enabled finding the best model that represented consensus information among the whole dataset. By identifying minimally overlapping high score models and calculating consensus weighting among them, the common features in the training dataset were captured. Besides the Erlotinib and Sorafenib case studies described in the current work, the method was further validated through our participation in the NCI-DREAM challenge. The NCI-DREAM challenge was a community effort comprised of 44 teams to predict drug-sensitivity to 28 drugs [41].

### Cell line data derived drug sensitivity models that accurately predict patients’ PFS--a translational medicine case study

A translational medicine case study consisting of generating predictive models of Erlotinib and Sorafenib sensitivity from a cell line panel dataset, followed by model validation in a clinical setting is presented. The models predict clear clinical benefits: for example, the Erlotinib drug sensitivity model separated marker-sensitive and marker-resistant patient groups with the former demonstrating twice as long survival than the latter (2 months difference with a Hazard ratio of 0.43, Fig 4A).

The signature gene set comprising the Erlotinib predictive model correctly captured Erlotinib’s mechanism of action as an EGFR inhibitor (Fig 2A). Moreover, the reconstructed signaling network for the Erlotinib signature featured several highly expressed growth factors linked to Erlotinib resistance (Fig 2A, please see S3 Fig and S1 Text for details) [42, 43]. Similarly, the network built for the Sorafenib signature was also clearly linked to its mechanism of action (Fig 2B, please see S4 Fig and S1 Text for details).

The training and testing datasets and the end-points were different: the models were built from a 2D in vitro cell line panel with IC50’s representing drug sensitivity and the validation study was conducted on primary tumors from BATTLE clinical trial patients with progress free survival (PFS) as the clinical end-point. Moreover, the training data (Oncopanel) covers a mixture of cancer indications, while non-small cell lung cancer (NSCLC) was the only cancer indication in the BATTLE trial. Also, the signatures were generated on the Affymetrix U133plus2 platform and tested on the data generated on Affymetrix Human Gene 1.0 ST platform. Despite all these differences, the cell line derived Erlotinib and Sorafenib sensitivity models predicted BATTLE trial PFS outcomes with high accuracy of 84% and 79%, respectively (Fig 3).

Given the fact the Erlotinib is an EGFR inhibitor while Sorafenib has multiple tyrosine kinase receptors as targets, one would expect that model specificity between Erlotinib and Sorafenib would be difficult to achieve. Strikingly, the Erlotinib and Sorafenib drug sensitivity models were drug specific, i.e. the Erlotinib model failed to predict Sorafenib patients’ PFS and vice versa (Figs 3 and 4).

The drug sensitivity models were used to predict percentage of drug sensitive samples per cancer indication for a large number of cancer expression datasets in various indications. The predicted Erlotinib or Sorafenib sensitive cancer indications were generally consistent with known FDA approvals on Erlotinib or Sorafenib (Table 1). Therefore, it indicates that cell-line derived models could be used to infer potential drug sensitive cancer indications. To the best of our knowledge, this is the first report on successful application of a predictive model generated from a mixture of cancer cell line screen dataset to predict potential drug-sensitive cancer indications.

## Methods

### Baseline gene expression datasets and data preprocess

Among the 240 Eurofin cancer cell lines, baseline gene expression data was downloaded from 170 cell lines from ArrayExpress (E-MTAB-37). The remaining 70 cell lines gene expression data was purchased from Eurofins (https://www.eurofinspanlabs.com). For testing the Erlotinib predictive model, the BATTLE trial patients’ baseline gene expression [27] (GSE33072) from the GEO database was downloaded. A RMA normalization was done on each dataset separately, using Affy package from Bioconductor [44].

Since gene expression data was generated from different microarray platforms, it is necessary to normalize them before model training/testing. A “U133PlusVsHuGene_BestMatch” file was downloaded from Affymetrix website (http://www.affymetrix.com) and was used to identify good matching probesets in Affymetrix U133plus2 array (for training dataset) and in Human Gene 1.0 ST array (for testing dataset). Based on the observations from both MAQC II project [16, 45] and other reports [46, 47], gene expression data from different studies and/or platforms can be first normalized and then analyzed. In the current work, a quantile-based reference-RMA normalization approach [47] was used to normalize the testing dataset against the training dataset.

### Compound sensitivity screen data on OncoPanel cancer cell line panel

Erlotinib and Sorafenib sensitivity screen data were purchased from Eurofins (https://www.eurofinspanlabs.com). There are 213 cell line IC50 data on both Erlotinib and Sorafenib datasets.

Two-layers of cell line data QC were conducted that reduced the number of cell lines from 213 to 183 before model training and testing. The first layer involved curve-fitting on dose-response data for each compound. The following criteria were used to QC each compound’s dose-response curve: (1). Cell lines were removed if there were three or more missing data points on the dose-response curve; (2). Cell lines were removed if there were four or more data points with CV > 30%; (3). Cell lines where the dose-response curve displays unusual behavior (e.g. has a stronger inhibitory effect at lower concentrations then at higher concentrations) were removed; (4). Cell lines that only demonstrate inhibitory effects at the highest tested concentration were removed. The second layer of QC evaluates whether cell line screen results actually reflect a drug-treatment effect. Clustering analysis was conducted among a group of internal and standard of care compounds. Compounds that have similar mechanism of action were clustered together, suggesting that cell line screen data actually reflects real drug-treatment effect and therefore could be possible to identify drug-sensitive predictive models from cell line data (data not shown).

### The PLSR based modeling framework

We designed and implemented a PLSR based modeling framework on drug-sensitivity predictive model building. More details on the PLSR method and its biological applications have been previously published [24–26]. We selected PLS regression from various regression approaches based on the following reasons: (1). PLS regression project both independent variables (gene expression values) and dependent variables (IC50s) in new directions to find maximum co-variance between these variables, while approaches like PCA analysis only capture variance in the independent variable space; (2). PLS regression is not subject to the multiple collinearity issue; (3). PLSR works very well on high number of independent variables vs limited number of dependent variables (number of feature genes >> number of samples), which fits very well with gene signature identification for cell line screens and clinical patient samples.

In the current work, we use the “pls” package from R (version 3.0.2) for PLS regression. The loading numbers from first and second PLS components were used to calculate gene weights as sqrt(PLS1^2 + PLS2^2). The gene weights were used to compare top splitting cases and forward feature selection steps.

### Reconstruction of causal network from signature genes

To reveal functional connectivity between the genes from Sorafenib and Erlotinib predictive models, we reconstructed the causal molecular network by connecting these genes via canonical molecular pathways. The network was built in several steps.

First, in addition to the predictive models’ genes, we identified “topologically significant” genes for the signature genes. The algorithm of selection of topologically-significant direct regulators, or “overconnected” genes, is described in [48] and remote regulators, or “hidden” nodes, is described in [49, 50].

Next, we selected molecular pathways significantly enriched with both genes of predictive signature and topologically significant genes using enrichment analysis in the Pathway Maps ontology in MetaCore (Thomson Reuters).

Finally, the identified enriched molecular pathways were manually combined in the causal molecular network. The resulting network included both sensitivity (negative correlation of expression with IC50) and resistance-specific (positive correlation of expression with IC50) genes. The networks were further populated with direct interactions between proteins encoded by signature genes. Also, we included genes with genetic alterations correlated with the drug response phenotype in OncoPanel cancer cell line panel in the reconstructed causal networks.

## Supporting Information

S1 Fig 183 cancer cell lines were selected to represent both haematopietic and solid tumor types to build Erlotinib or Sorafenib predictive models. (TIF)

S2 Fig Comparing signature gene’s contribution among top not-significantly-overlapped models. Pairwise comparison was done among top performing models, as well as with the consensus model. The numbers in the lower left part of the figure are PLSR model derived loading values for individual genes, and the numbers in the top right part of the figure are Pearson correlations between models.
(TIF)

S3 Fig Reconstructed network derived from Erlotinib signature genes. The network was reconstructed from canonical signaling pathways regulated by signature genes and signature specific direct interaction network. Sensitivity-specific signature genes are highlighted with blue thermometers, resistance-specific genes are red thermometers; topologically significant genes are highlighted with yellow thermometers. White starts in object images mark groups of proteins.
(TIF)

S4 Fig Reconstructed network derived from Sorafenib signature genes. The network was reconstructed from canonical signaling pathways regulated by signature genes and signature specific direct interaction network. Sensitivity-specific signature genes are highlighted with blue thermometers, resistance-specific genes are highlighted with red thermometers; topologically significant genes are highlighted with yellow thermometers. White starts in object images mark groups of proteins.
(TIF)

S5 Fig Erlotinib and Sorafenib IC50 distributions on Oncopanel cancer cell line panel. (A) Erlotinib IC50 distribution on Oncopanel cancer cell line panel; (B). Sorafenib IC50 distribution on Oncopanel cancer cell line panel. The top figure was for the whole Ricerca cell line panel and the bottom figure was after removing data for middle one-third IC50s.
(TIF)

S6 Fig Representative example of random validation and balance validation on Sorafenib model training. Red points were top performing models on 1000 random splits on this balanced split, based on both AUC and correlation measures.
(TIF)

S7 Fig Distribution of PLSR model predicted scores (log_2_(IC50)) for Erlotinib and Sorafenib. Red vertical line was cutoffs selected to separate drug sensitive vs resistant cases for each drug.
(TIF)

S8 Fig Progression Free Survival for patients in Erlotinib or Sorafenib arms of the BATTLE clinical trials. Red vertical line was cutoffs selected to separate patients into responder and non-responder sub-groups for each drug.
(TIF)

S1 Table The 51 Erlotinib signature genes. A raw p-value of each gene was calculated during feature selection step based on the fitted mean and standard deviation from the permutation data (see Methods in the main text section). Adjusted p-value was calculated using Benjamini-Hochberg control of false discovery rate.
(DOCX)

S2 Table The 113 gene Sorafenib signature genes. A raw p-value of each gene was calculated during feature selection step based on the fitted mean and standard deviation from the permutation data (see Methods in the main text section). Adjusted p-value was calculated using Benjamini-Hochberg control of false discovery rate.
(DOCX)

S3 Table Association analysis of genetic events and drug response. Pearson’s correlation coefficient was calculated for a vector of log2(IC50) and a vector of genetic events. Positive value of correlation for a given gene was interpreted as correlation with resistance. Negative value can was interpreted as an association with sensitivity. Correlation P-values were calculated by a permutation test with 1000 shuffles. Fisher’s exact test was used for association analysis between resistance/sensitivity classes and genetic events. “R” stands for resistance, “S” stands for sensitivity.
(DOCX)

S1 Text Description of causal models reconstructed from Erlotinib and Sorafenib predictive models. Description and discussion of reconstructed causal models omitted from main manuscript and shown in S3 and S4 Fig and Fig 2 in the main manuscript section.
(DOCX)

S2 Text Association analysis of genetic events. Additional results and methods omitted from the main manuscript. We provide results of association analysis between genetic events and drug response supporting major observations in the manuscript.
(DOCX)

## References

[1] Reinhold WC, Sunshine M, Liu H, Varma S, Kohn KW, Morris J, et al CellMiner: A Web-Based Suite of Genomic and Pharmacologic Tools to Explore Transcript and Drug Patterns in the NCI-60 Cell Line Set. Cancer Res. 2012;72(14):3499–511. Epub 2012/07/18. doi: 0008-5472.CAN-12-1370 [pii] 10.1158/0008-5472.CAN-12-1370 22802077 PMC3399763

[2] Bussey KJ, Chin K, Lababidi S, Reimers M, Reinhold WC, Kuo WL, et al Integrating data on DNA copy number with gene expression levels and drug sensitivities in the NCI-60 cell line panel. Mol Cancer Ther. 2006;5(4):853–67. Epub 2006/05/02. doi: 5/4/853 [pii] 10.1158/1535-7163.MCT-05-0155 16648555 PMC2733874

[3] Lee JK, Havaleshko DM, Cho H, Weinstein JN, Kaldjian EP, Karpovich J, et al A strategy for predicting the chemosensitivity of human cancers and its application to drug discovery. Proc Natl Acad Sci U S A. 2007;104(32):13086–91. Epub 2007/08/02. doi: 0610292104 [pii] 10.1073/pnas.0610292104 17666531 PMC1941805

[4] Shoemaker RH, Monks A, Alley MC, Scudiero DA, Fine DL, McLemore TL, et al Development of human tumor cell line panels for use in disease-oriented drug screening. Prog Clin Biol Res. 1988;276:265–86. Epub 1988/01/01. . 3051021

[5] Weinstein JN, Myers TG, O'Connor PM, Friend SH, Fornace AJ Jr., Kohn KW, et al An information-intensive approach to the molecular pharmacology of cancer. Science. 1997;275(5298):343–9. Epub 1997/01/17. . 8994024 10.1126/science.275.5298.343

[6] Lee JK, Bussey KJ, Gwadry FG, Reinhold W, Riddick G, Pelletier SL, et al Comparing cDNA and oligonucleotide array data: concordance of gene expression across platforms for the NCI-60 cancer cells. Genome Biol. 2003;4(12):R82 Epub 2003/12/09. doi: 10.1186/gb-2003-4-12-r82 gb-2003-4-12-r82 [pii] 14659019 10.1186/gb-2003-4-12-r82 PMC329421

[7] Nishizuka S, Charboneau L, Young L, Major S, Reinhold WC, Waltham M, et al Proteomic profiling of the NCI-60 cancer cell lines using new high-density reverse-phase lysate microarrays. Proc Natl Acad Sci U S A. 2003;100(24):14229–34. Epub 2003/11/19. doi: 10.1073/pnas.2331323100 2331323100 [pii] 14623978 10.1073/pnas.2331323100 PMC283574

[8] Reinhold WC, Reimers MA, Maunakea AK, Kim S, Lababidi S, Scherf U, et al Detailed DNA methylation profiles of the E-cadherin promoter in the NCI-60 cancer cells. Mol Cancer Ther. 2007;6(2):391–403. Epub 2007/02/03. doi: 1535-7163.MCT-06-0609 [pii] 10.1158/1535-7163.MCT-06-0609 . 17272646

[9] Blower PE, Verducci JS, Lin S, Zhou J, Chung JH, Dai Z, et al MicroRNA expression profiles for the NCI-60 cancer cell panel. Mol Cancer Ther. 2007;6(5):1483–91. Epub 2007/05/08. doi: 1535-7163.MCT-07-0009 [pii] 10.1158/1535-7163.MCT-07-0009 . 17483436

[10] Fagan A, Culhane AC, Higgins DG. A multivariate analysis approach to the integration of proteomic and gene expression data. Proteomics. 2007;7(13):2162–71. Epub 2007/06/06. 10.1002/pmic.200600898 . 17549791

[11] Ikediobi ON, Davies H, Bignell G, Edkins S, Stevens C, O'Meara S, et al Mutation analysis of 24 known cancer genes in the NCI-60 cell line set. Mol Cancer Ther. 2006;5(11):2606–12. Epub 2006/11/08. doi: 1535-7163.MCT-06-0433 [pii] 10.1158/1535-7163.MCT-06-0433 17088437 PMC2705832

[12] Garnett MJ, Edelman EJ, Heidorn SJ, Greenman CD, Dastur A, Lau KW, et al Systematic identification of genomic markers of drug sensitivity in cancer cells. Nature. 2012;483(7391):570–5. Epub 2012/03/31. doi: 10.1038/nature11005 nature11005 [pii] 22460902 10.1038/nature11005 PMC3349233

[13] Barretina J, Caponigro G, Stransky N, Venkatesan K, Margolin AA, Kim S, et al The Cancer Cell Line Encyclopedia enables predictive modelling of anticancer drug sensitivity. Nature. 2012;483(7391):603–7. Epub 2012/03/31. doi: 10.1038/nature11003 nature11003 [pii] 22460905 10.1038/nature11003 PMC3320027

[14] van de Vijver MJ, He YD, van't Veer LJ, Dai H, Hart AA, Voskuil DW, et al A gene-expression signature as a predictor of survival in breast cancer. N Engl J Med. 2002;347(25):1999–2009. Epub 2002/12/20. 10.1056/NEJMoa021967 347/25/1999 [pii] . 12490681

[15] Paik S, Shak S, Tang G, Kim C, Baker J, Cronin M, et al A multigene assay to predict recurrence of tamoxifen-treated, node-negative breast cancer. N Engl J Med. 2004;351(27):2817–26. Epub 2004/12/14. doi: NEJMoa041588 [pii] 10.1056/NEJMoa041588 . 15591335

[16] Shi L, Campbell G, Jones WD, Campagne F, Wen Z, Walker SJ, et al The MicroArray Quality Control (MAQC)-II study of common practices for the development and validation of microarray-based predictive models. Nat Biotechnol. 2010;28(8):827–38. Epub 2010/08/03. doi: nbt.1665 [pii] 10.1038/nbt.1665 20676074 PMC3315840

[17] Zhan F, Huang Y, Colla S, Stewart JP, Hanamura I, Gupta S, et al The molecular classification of multiple myeloma. Blood. 2006;108(6):2020–8. Epub 2006/05/27. doi: blood-2005-11-013458 [pii] 10.1182/blood-2005-11-013458 16728703 PMC1895543

[18] Shaughnessy JD Jr., Zhan F, Burington BE, Huang Y, Colla S, Hanamura I, et al A validated gene expression model of high-risk multiple myeloma is defined by deregulated expression of genes mapping to chromosome 1. Blood. 2007;109(6):2276–84. Epub 2006/11/16. doi: blood-2006-07-038430 [pii] 10.1182/blood-2006-07-038430 . 17105813

[19] Zhan F, Barlogie B, Mulligan G, Shaughnessy JD Jr., Bryant B. High-risk myeloma: a gene expression based risk-stratification model for newly diagnosed multiple myeloma treated with high-dose therapy is predictive of outcome in relapsed disease treated with single-agent bortezomib or high-dose dexamethasone. Blood. 2008;111(2):968–9. Epub 2008/01/10. doi: 111/2/968 [pii] 10.1182/blood-2007-10-119321 18182586 PMC2200846

[20] Decaux O, Lode L, Magrangeas F, Charbonnel C, Gouraud W, Jezequel P, et al Prediction of survival in multiple myeloma based on gene expression profiles reveals cell cycle and chromosomal instability signatures in high-risk patients and hyperdiploid signatures in low-risk patients: a study of the Intergroupe Francophone du Myelome. J Clin Oncol. 2008;26(29):4798–805. Epub 2008/07/02. 10.1200/JCO.2007.13.8545 JCO.2007.13.8545 [pii] . 18591550

[21] Mulligan G, Mitsiades C, Bryant B, Zhan F, Chng WJ, Roels S, et al Gene expression profiling and correlation with outcome in clinical trials of the proteasome inhibitor bortezomib. Blood. 2007;109(8):3177–88. Epub 2006/12/23. doi: blood-2006-09-044974 [pii] 10.1182/blood-2006-09-044974 . 17185464

[22] Lazar C, Meganck S, Taminau J, Steenhoff D, Coletta A, Molter C, et al Batch effect removal methods for microarray gene expression data integration: a survey. Briefings in bioinformatics. 2013;14(4):469–90. Epub 2012/08/02. 10.1093/bib/bbs037 . 22851511

[23] Lazar C, Taminau J, Meganck S, Steenhoff D, Coletta A, Solis DY, et al GENESHIFT: a nonparametric approach for integrating microarray gene expression data based on the inner product as a distance measure between the distributions of genes. IEEE/ACM transactions on computational biology and bioinformatics / IEEE, ACM. 2013;10(2):383–92. Epub 2013/08/10. 10.1109/TCBB.2013.12 . 23929862

[24] Wold S, Sjostrom M, Eriksson L. PLS-regression: a basic tool of chemometrics. Chemometrics and Intelligent Laboratory Systems. 2001;58:109–30.

[25] Janes KA, Gaudet S, Albeck JG, Nielsen UB, Lauffenburger DA, Sorger PK. The response of human epithelial cells to TNF involves an inducible autocrine cascade. Cell. 2006;124(6):1225–39. Epub 2006/03/28. doi: S0092-8674(06)00292-3 [pii] 10.1016/j.cell.2006.01.041 . 16564013

[26] Lindgren F, Geladi P, Wold S. The kernel algorithm for PLS. J Chemometrics. 1993;7:45–59.

[27] Kim ES, Herbst RS, Wistuba II, Lee JJ, Blumenschein GR Jr., Tsao A, et al The BATTLE trial: personalizing therapy for lung cancer. Cancer Discov. 2011;1(1):44–53. Epub 2012/05/16. doi: 2159-8274.CD-10-0010 [pii] 10.1158/2159-8274.CD-10-0010 . 22586319 PMC4211116

[28] Benjamini Y, Hochberg Y. The control of the false discovery rate in multiple testing under dependency. Annals of Statistics. 1995;29:1165–88.

[29] Chun H, Keles S. Expression quantitative trait loci mapping with multivariate sparse partial least squares regression. Genetics. 2009;182(1):79–90. Epub 2009/03/10. 10.1534/genetics.109.100362 19270271 PMC2674843

[30] Chun H, Keles S. Sparse partial least squares regression for simultaneous dimension reduction and variable selection. Journal of the Royal Statistical Society Series B, Statistical methodology. 2010;72(1):3–25. Epub 2010/01/29. 10.1111/j.1467-9868.2009.00723.x 20107611 PMC2810828

[31] Le Cao KA, Boitard S, Besse P. Sparse PLS discriminant analysis: biologically relevant feature selection and graphical displays for multiclass problems. BMC Bioinformatics. 2011;12:253. Epub 2011/06/23. doi: 1471-2105-12-253 [pii] 10.1186/1471-2105-12-253 21693065 PMC3133555

[32] Le Cao KA, Martin PG, Robert-Granie C, Besse P. Sparse canonical methods for biological data integration: application to a cross-platform study. BMC Bioinformatics. 2009;10:34 Epub 2009/01/28. 10.1186/1471-2105-10-34 19171069 PMC2640358

[33] Witten DM, Tibshirani RJ. Extensions of sparse canonical correlation analysis with applications to genomic data. Statistical applications in genetics and molecular biology. 2009;8:Article28 Epub 2009/07/04. 10.2202/1544-6115.1470 19572827 PMC2861323

[34] Demmel J, Kahan W. Accurate Singular-Values of Bidiagonal Matrices. Siam J Sci Stat Comp. 1990;11(5):873–912. 10.1137/0911052 WOS:A1990DU98400005.

[35] Bessarabova M, Ishkin A, JeBailey L, Nikolskaya T, Nikolsky Y. Knowledge-based analysis of proteomics data. BMC Bioinformatics. 2012;13(Suppl 16):S13 10.1186/1471-2105-13-S16-S13 23176192 PMC3489533

[36] Perakslis ED, Van Dam J, Szalma S. How informatics can potentiate precompetitive open-source collaboration to jump-start drug discovery and development. Clin Pharmacol Ther. 2010;87(5):614–6. Epub 2010/04/09. 10.1038/clpt.2010.21 clpt201021 [pii] . 20376001

[37] McCall MN, Irizarry RA. Thawing Frozen Robust Multi-array Analysis (fRMA). BMC Bioinformatics. 2011;12:369 Epub 2011/09/20. 10.1186/1471-2105-12-369 1471-2105-12-369 [pii] 21923903 PMC3180392

[38] Virtanen C, Ishikawa Y, Honjoh D, Kimura M, Shimane M, Miyoshi T, et al Integrated classification of lung tumors and cell lines by expression profiling. Proc Natl Acad Sci U S A. 2002;99(19):12357–62. 10.1073/pnas.192240599 12218176 PMC129449

[39] Carlson MW, Iyer VR, Marcotte EM. Quantitative gene expression assessment identifies appropriate cell line models for individual cervical cancer pathways. BMC genomics. 2007;8:117 10.1186/1471-2164-8-117 17493265 PMC1878486

[40] Blumenschein GR Jr., Saintigny P, Liu S, Kim ES, Tsao AS, Herbst RS, et al Comprehensive biomarker analysis and final efficacy results of sorafenib in the BATTLE trial. Clin Cancer Res. 2013;19(24):6967–75. Epub 2013/10/30. 10.1158/1078-0432.CCR-12-1818 1078-0432.CCR-12-1818 [pii] 24166906 PMC3905243

[41] Costello JC, Heiser LM, Georgii E, Gonen M, Menden MP, Wang NJ, et al A community effort to assess and improve drug sensitivity prediction algorithms. Nat Biotechnol. 2014;32(12):1202–12. Epub 2014/06/02. 10.1038/nbt.2877 . 24880487 PMC4547623

[42] Bar J, Onn A. Overcoming molecular mechanisms of resistance to first-generation epidermal growth factor receptor tyrosine kinase inhibitors. Clinical lung cancer. 2012;13(4):267–79. 10.1016/j.cllc.2011.09.001 . 22154113

[43] Wheeler DL, Dunn EF, Harari PM. Understanding resistance to EGFR inhibitors-impact on future treatment strategies. Nature reviews Clinical oncology. 2010;7(9):493–507. 10.1038/nrclinonc.2010.97 20551942 PMC2929287

[44] Bolstad BM, Irizarry RA, Astrand M, Speed TP. A comparison of normalization methods for high density oligonucleotide array data based on variance and bias. Bioinformatics. 2003;19(2):185–93. Epub 2003/01/23. . 12538238 10.1093/bioinformatics/19.2.185

[45] Luo J, Schumacher M, Scherer A, Sanoudou D, Megherbi D, Davison T, et al A comparison of batch effect removal methods for enhancement of prediction performance using MAQC-II microarray gene expression data. Pharmacogenomics J. 2010;10(4):278–91. Epub 2010/08/03. doi: tpj201057 [pii] 10.1038/tpj.2010.57 20676067 PMC2920074

[46] McCall MN, Bolstad BM, Irizarry RA. Frozen robust multiarray analysis (fRMA). Biostatistics. 2010;11(2):242–53. 10.1093/biostatistics/kxp059 20097884 PMC2830579

[47] Katz S, Irizarry RA, Lin X, Tripputi M, Porter MW. A summarization approach for Affymetrix GeneChip data using a reference training set from a large, biologically diverse database. BMC Bioinformatics. 2006;7:464. Epub 2006/10/25. doi: 1471-2105-7-464 [pii] 10.1186/1471-2105-7-464 17059591 PMC1624855

[48] Nikolsky Y, Kirillov E, Zuev R, Rakhmatulin E, Nikolskaya T. Functional Analysis of OMICs Data and Small Molecule Compounds in an Integrated "Knowledge-Based" Platform. Methods Mol Biol. 2009;563:177–96. 10.1007/978-1-60761-175-2_10 WOS:000269615300010. 19597786

[49] Vellaichamy A, Dezso Z, JeBailey L, Chinnaiyan AM, Sreekumar A, Nesvizhskii AI, et al "Topological Significance" Analysis of Gene Expression and Proteomic Profiles from Prostate Cancer Cells Reveals Key Mechanisms of Androgen Response. Plos One. 2010;5(6). doi: ARTN e10936 10.1371/journal.pone.0010936 WOS:000278318700010. PMC2880599 20532174

[50] Dezso Z, Nikolsky Y, Nikolskaya T, Miller J, Cherba D, Webb C, et al Identifying disease-specific genes based on their topological significance in protein networks. Bmc Syst Biol. 2009;3. doi: Artn 36. 10.1186/1752-0509-3-36 WOS:000266001700001. PMC2678983 19309513

