# Supplementary figures and images for: Development of a Drug-Response Modeling Framework to Identify Cell Line Derived Translational Biomarkers That Can Predict Treatment Outcome to Erlotinib or Sorafenib
Source: PLoS One. 2015 Jun 24;10(6):e0130700. doi: 10.1371/journal.pone.0130700 (PMC4480971; doi:10.1371/journal.pone.0130700)

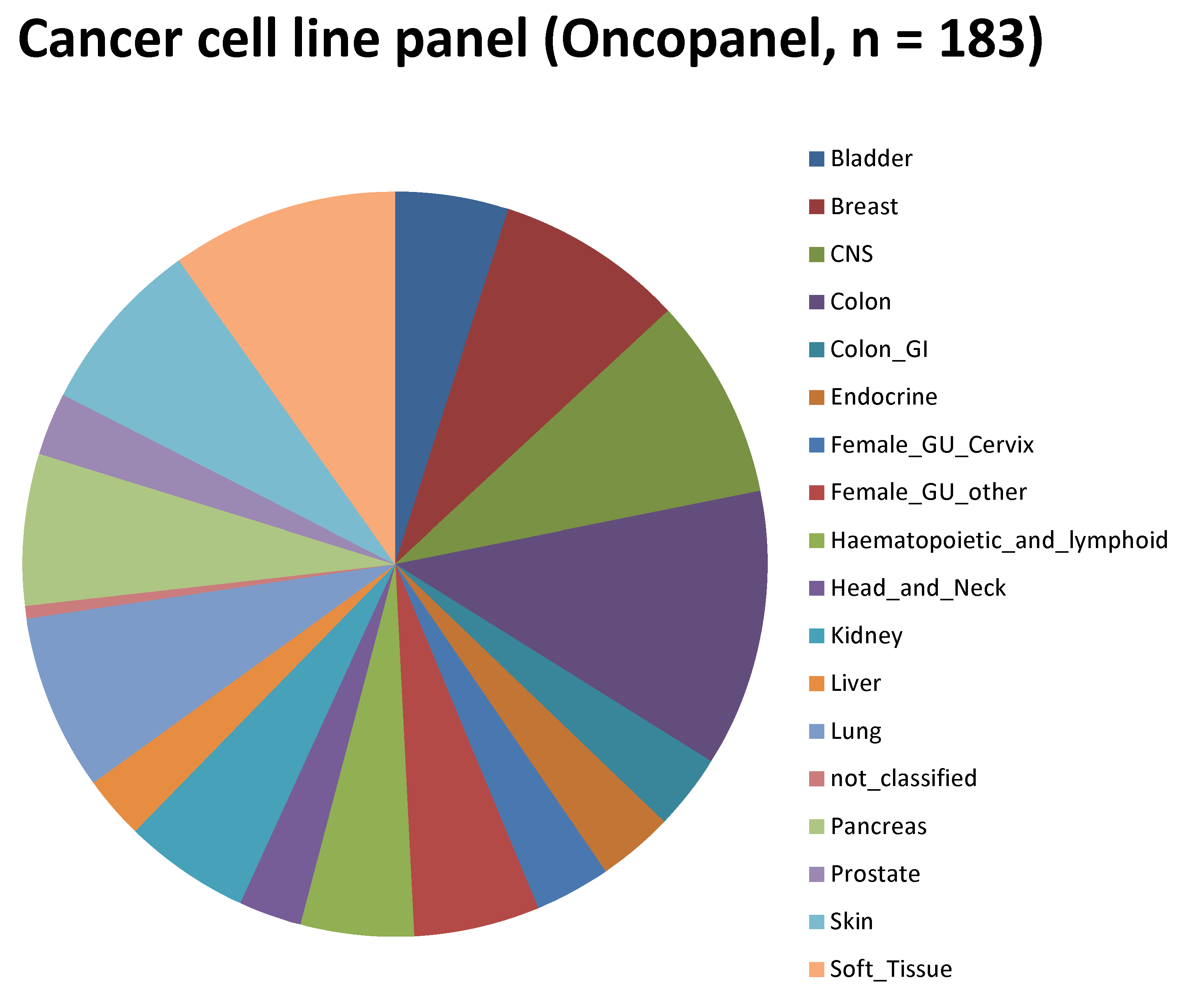

Supplement: S1 Fig — (TIF) [file pone.0130700.s001.tif]

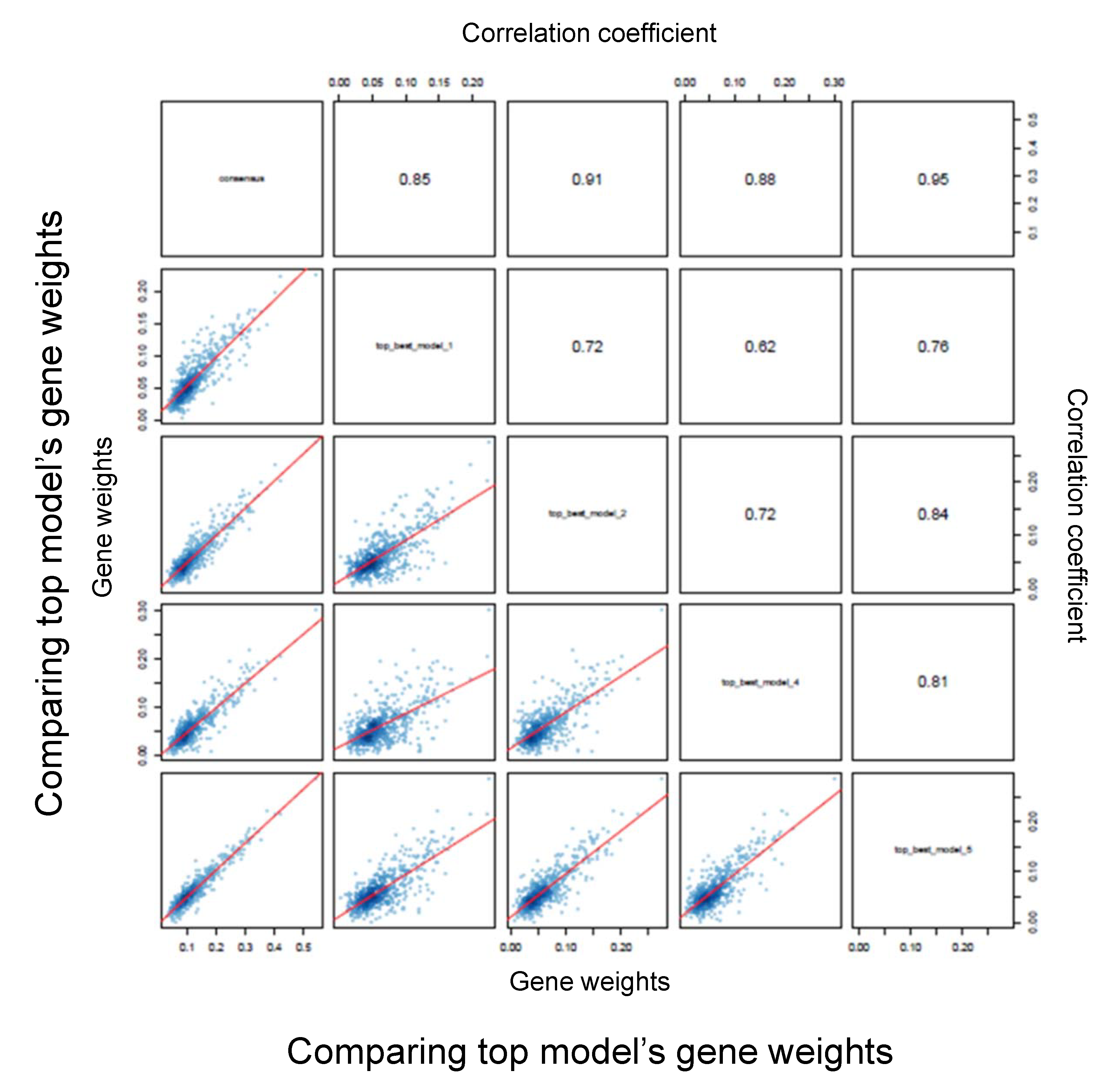

Supplement: S2 Fig — Pairwise comparison was done among top performing models, as well as with the consensus model. The numbers in the lower left part of the figure are PLSR model derived loading values for individual genes, and the numbers in the top right part of the figure are Pearson correlations between models. (TIF) [file pone.0130700.s002.tif]

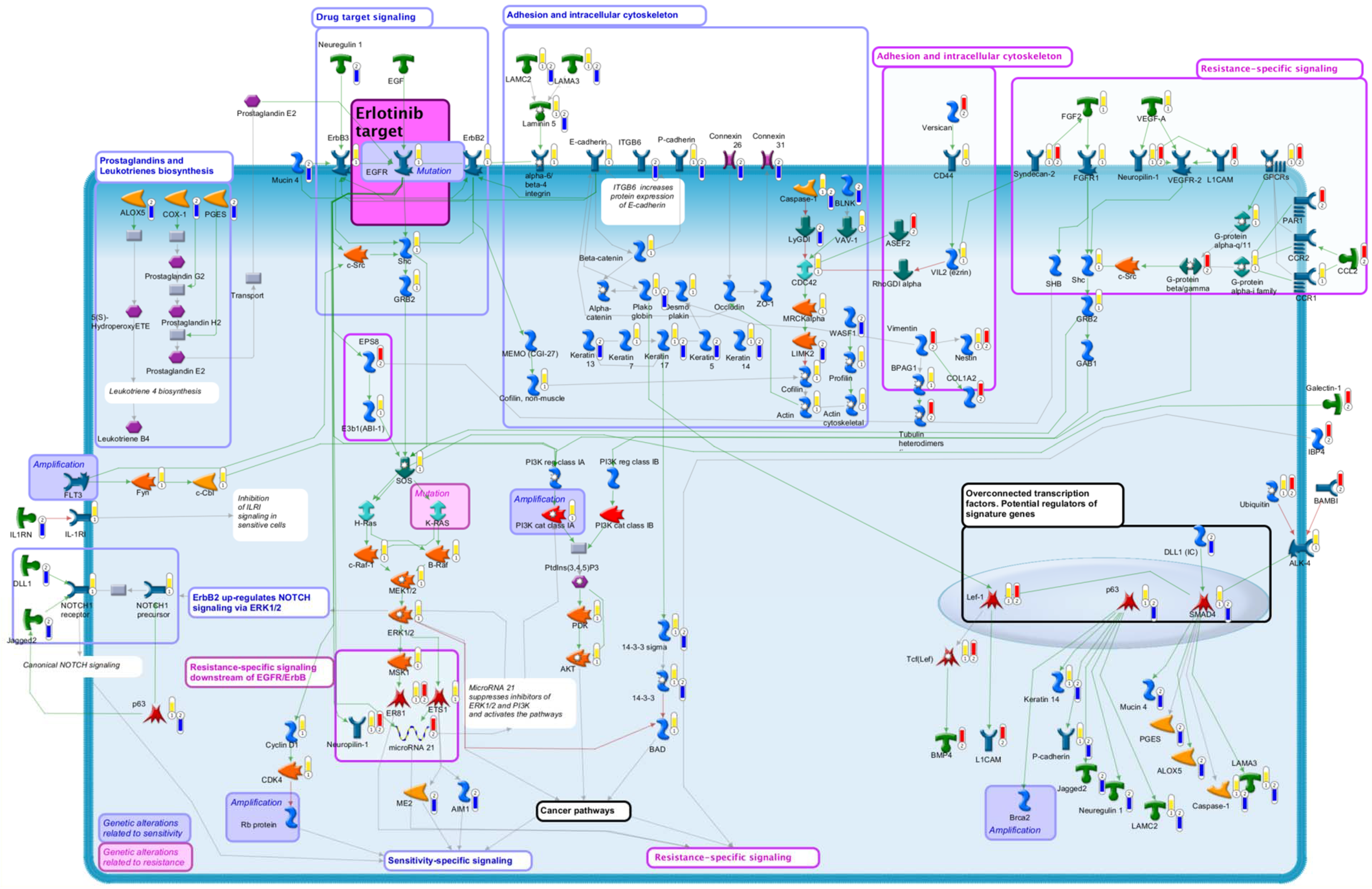

Supplement: S3 Fig — The network was reconstructed from canonical signaling pathways regulated by signature genes and signature specific direct interaction network. Sensitivity-specific signature genes are highlighted with blue thermometers, resistance-specific genes are red thermometers; topologically significant genes are highlighted with yellow thermometers. White starts in object images mark groups of proteins. (TIF) [file pone.0130700.s003.tif]

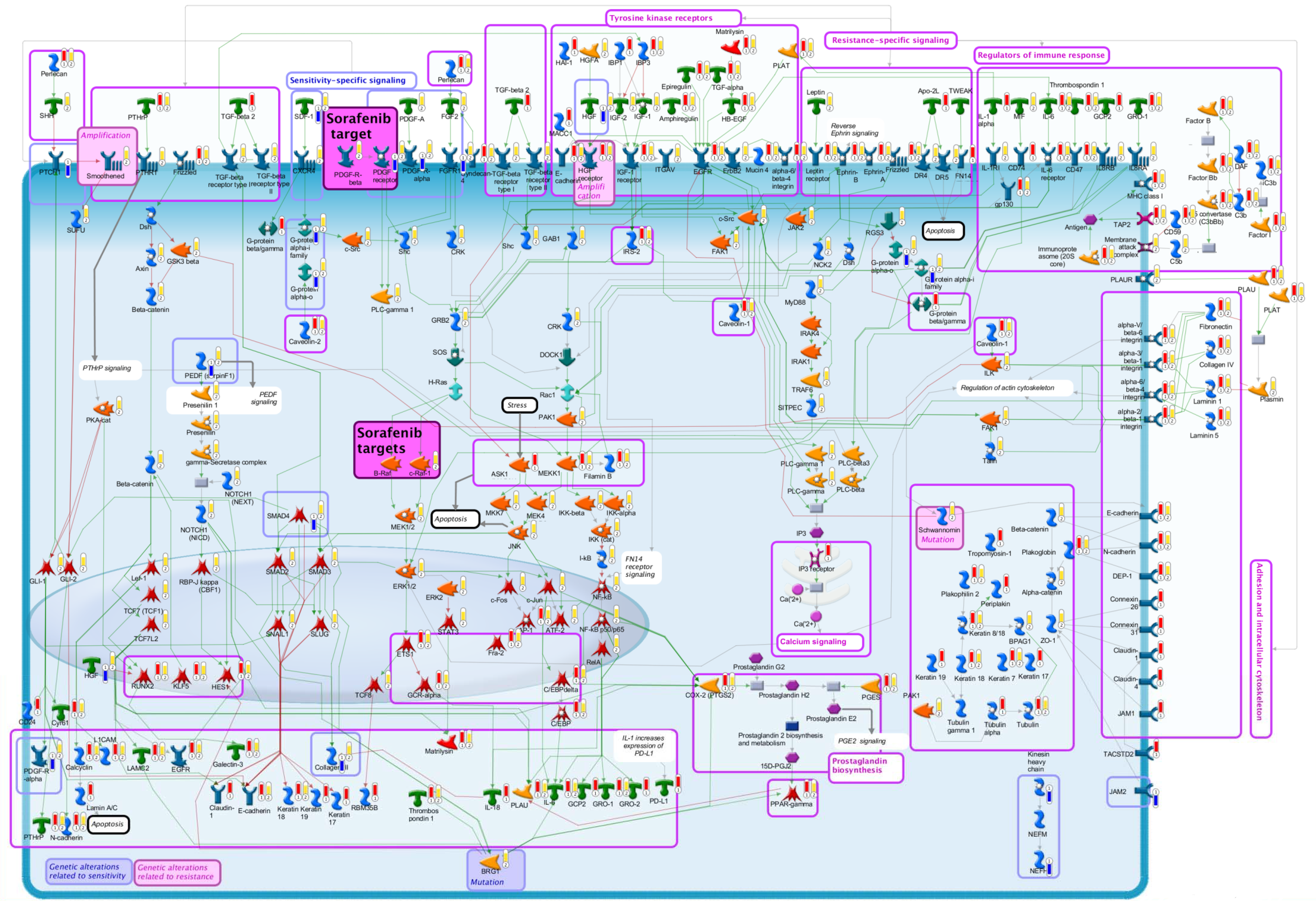

Supplement: S4 Fig — The network was reconstructed from canonical signaling pathways regulated by signature genes and signature specific direct interaction network. Sensitivity-specific signature genes are highlighted with blue thermometers, resistance-specific genes are highlighted with red thermometers; topologically significant genes are highlighted with yellow thermometers. White starts in object images mark groups of proteins. (TIF) [file pone.0130700.s004.tif]

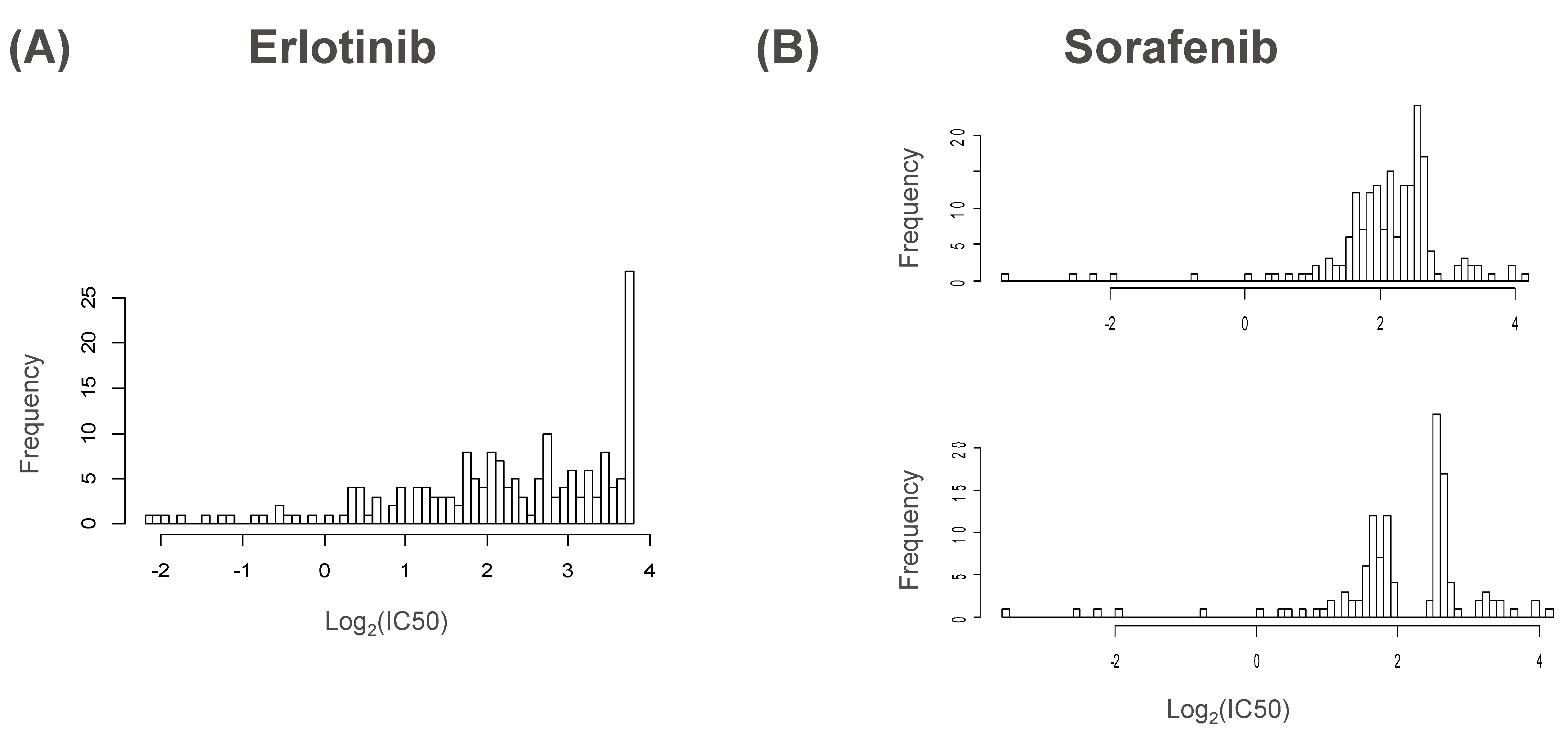

Supplement: S5 Fig — (A) Erlotinib IC50 distribution on Oncopanel cancer cell line panel; (B). Sorafenib IC50 distribution on Oncopanel cancer cell line panel. The top figure was for the whole Ricerca cell line panel and the bottom figure was after removing data for middle one-third IC50s. (TIF) [file pone.0130700.s005.tif]

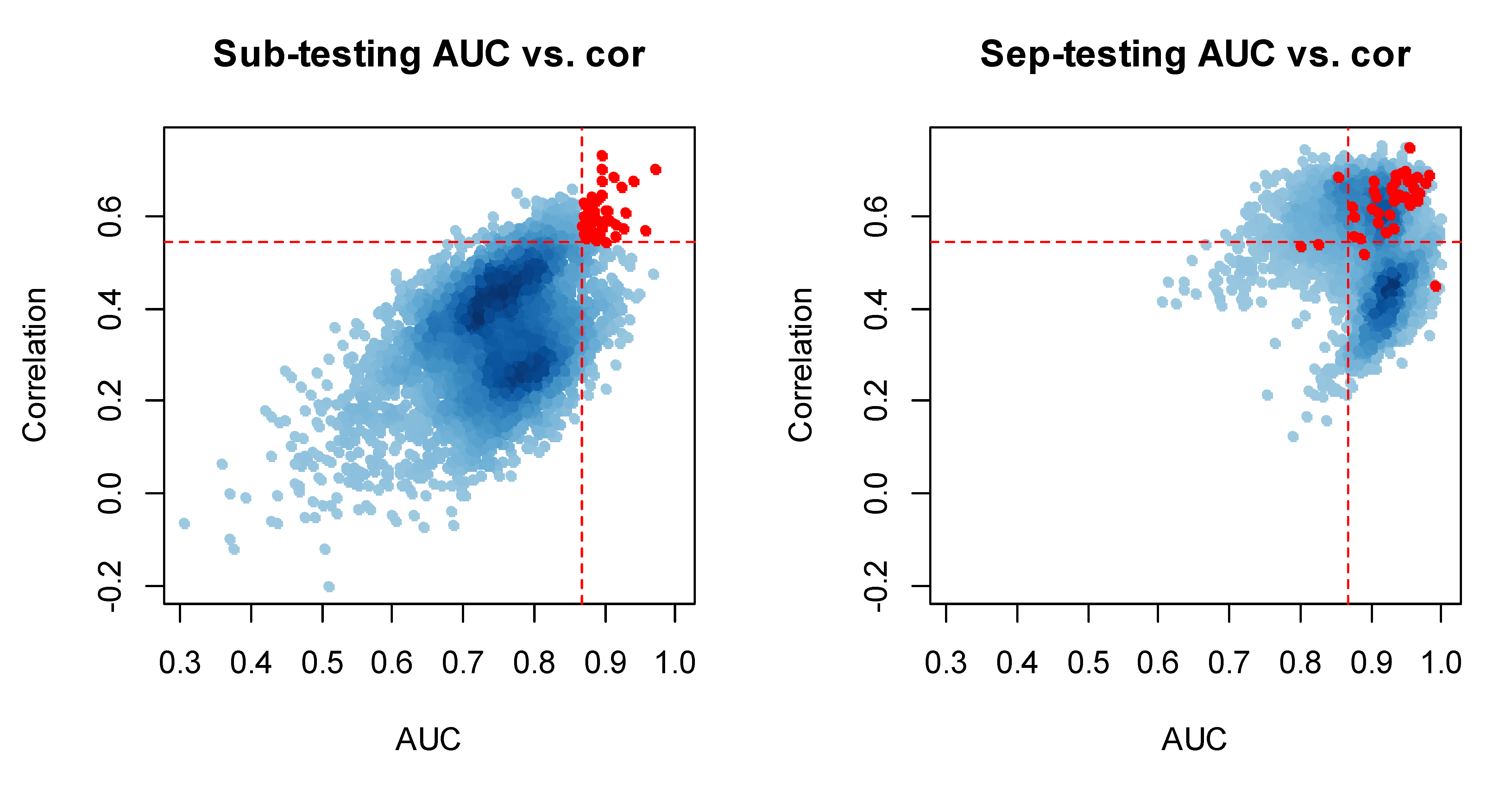

Supplement: S6 Fig — Red points were top performing models on 1000 random splits on this balanced split, based on both AUC and correlation measures. (TIF) [file pone.0130700.s006.tif]

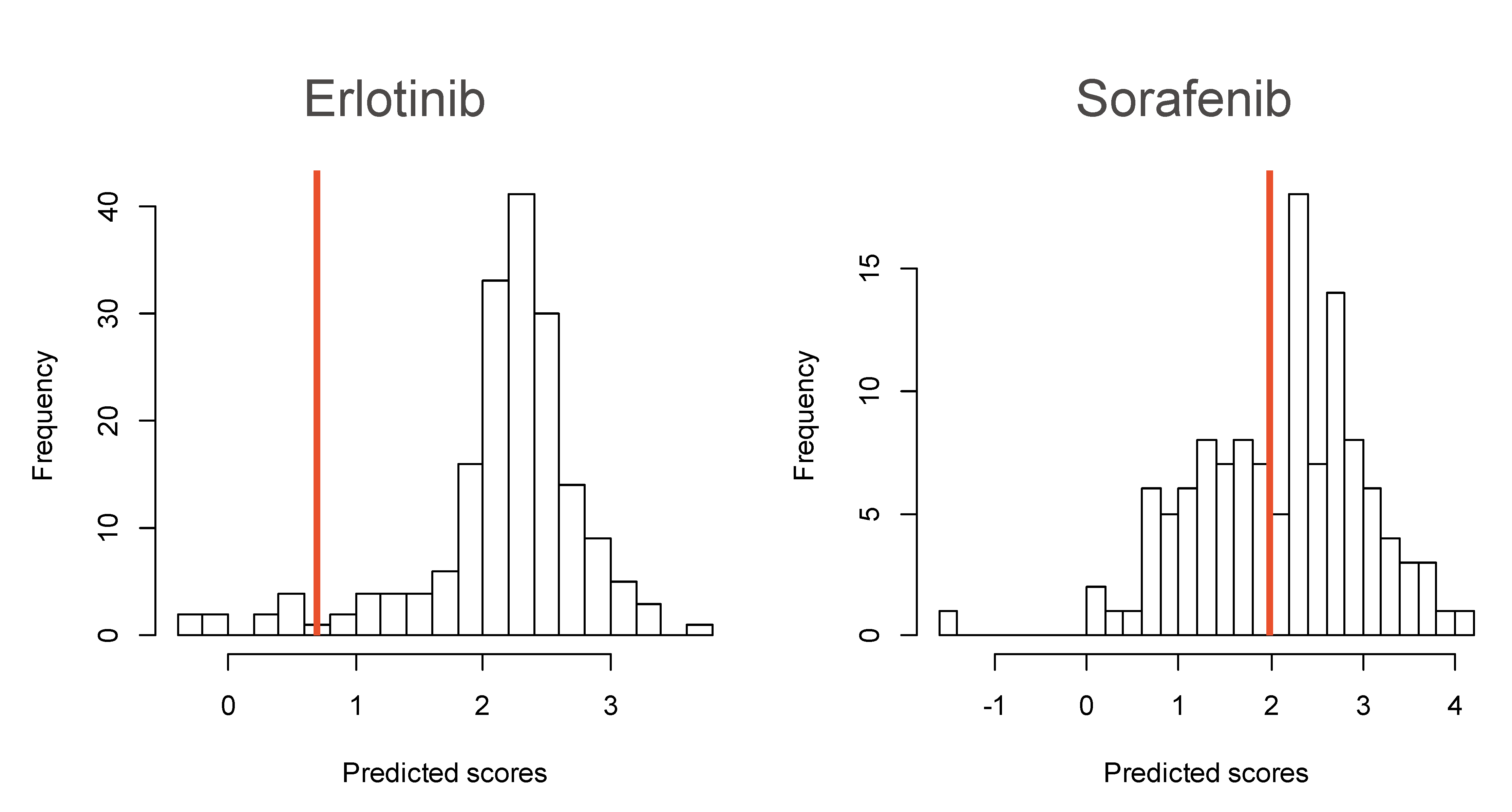

Supplement: S7 Fig — Red vertical line was cutoffs selected to separate drug sensitive vs resistant cases for each drug. (TIF) [file pone.0130700.s007.tif]

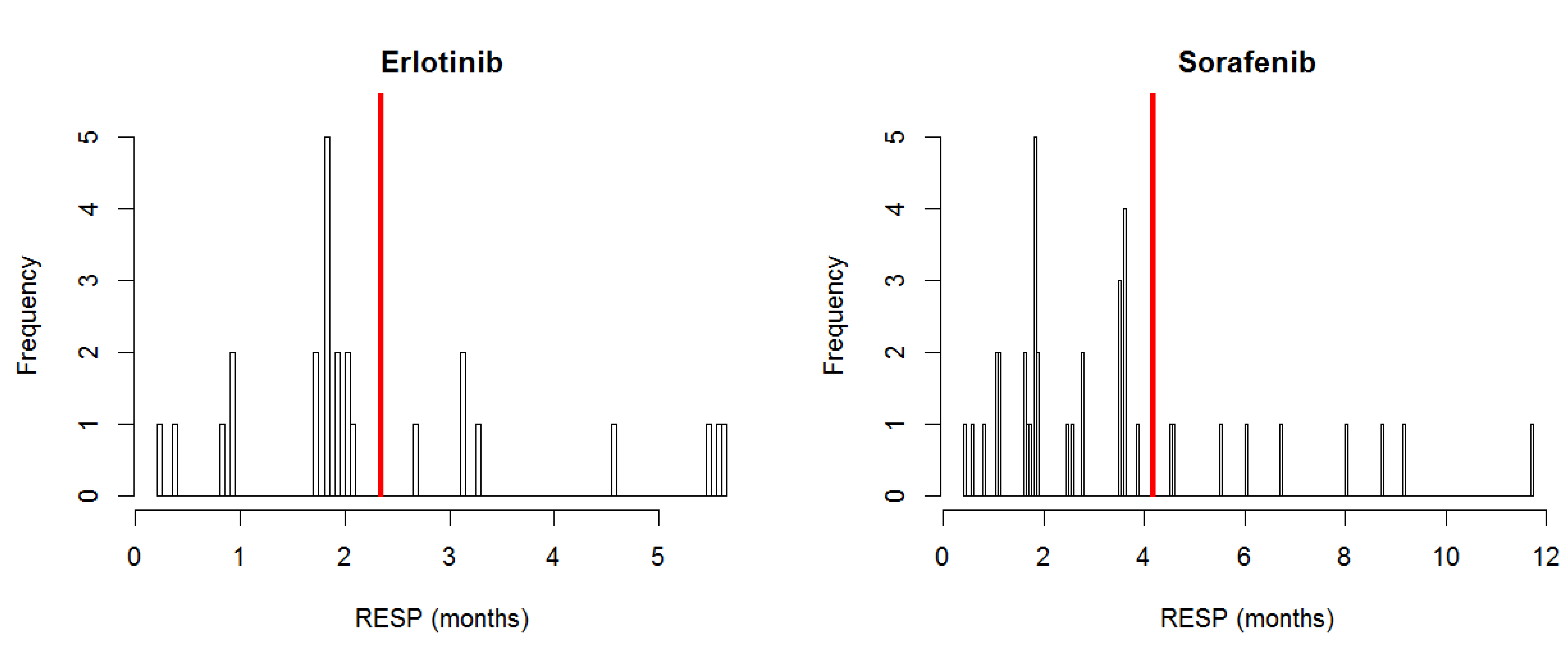

Supplement: S8 Fig — Red vertical line was cutoffs selected to separate patients into responder and non-responder sub-groups for each drug. (TIF) [file pone.0130700.s008.tif]
