# Supplementary material for: Development of a Drug-Response Modeling Framework to Identify Cell Line Derived Translational Biomarkers That Can Predict Treatment Outcome to Erlotinib or Sorafenib
Source: PLoS One. 2015 Jun 24;10(6):e0130700. doi: 10.1371/journal.pone.0130700 (PMC4480971; doi:10.1371/journal.pone.0130700)
Supplement: S1 Table — A raw p-value of each gene was calculated during feature selection step based on the fitted mean and standard deviation from the permutation data (see Methods in the main text section). Adjusted p-value was calculated using Benjamini-Hochberg control of false discovery rate. (DOCX) [file pone.0130700.s009.docx]

Table S1. The 51 Erlotinib signature genes

A raw p-value of each gene was calculated during feature selection step based on the fitted mean and standard deviation from the permutation data (see Methods in the main text section). Adjusted p-value was calculated using Benjamini-Hochberg control of false discovery rate.

| **ProbeSet ID** | **EntrezGene ID** | **Gene Symbol** | **Protein name** | **Correlation coefficient** | **p-value** | **p-value adjusted** |
| --- | --- | --- | --- | --- | --- | --- |
| 209863_s_at | 8626 | TP63 | Tumor protein 63 | -0.34 | 0.00 | 0.00 |
| 209351_at | 3861 | KRT14 | Keratin, type I cytoskeletal 14 | -0.31 | 0.00 | 0.01 |
| 201820_at | 3852 | KRT5 | Keratin, type II cytoskeletal 5 | -0.31 | 0.00 | 0.01 |
| 215813_s_at | 5742 | PTGS1 | Prostaglandin G/H synthase 1 | -0.29 | 0.00 | 0.01 |
| 224215_s_at | 28514 | DLL1 | Delta-like protein 1 | -0.28 | 0.00 | 0.02 |
| 207935_s_at | 3860 | KRT13 | Keratin, type I cytoskeletal 13 | -0.26 | 0.00 | 0.04 |
| 207655_s_at | 29760 | BLNK | B-cell linker protein | -0.25 | 0.00 | 0.05 |
| 212657_s_at | 3557 | IL1RN | Interleukin-1 receptor antagonist protein | -0.25 | 0.00 | 0.05 |
| 209397_at | 4200 | ME2 | NAD-dependent malic enzyme, mitochondrial | -0.23 | 0.00 | 0.07 |
| 212236_x_at | 3872 | KRT17 | Keratin, type I cytoskeletal 17 | -0.21 | 0.00 | 0.13 |
| 203256_at | 1001 | CDH3 | Cadherin-3 | -0.20 | 0.00 | 0.15 |
| 1552797_s_at | 150696 | PROM2 | Prominin-2 | -0.20 | 0.01 | 0.18 |
| 205490_x_at | 2707 | GJB3 | Gap junction beta-3 protein | -0.20 | 0.01 | 0.16 |
| 211368_s_at | 834 | CASP1 | Caspase-1 | -0.19 | 0.01 | 0.15 |
| 202267_at | 3918 | LAMC2 | Laminin subunit gamma-2 | -0.19 | 0.01 | 0.21 |
| 203726_s_at | 3909 | LAMA3 | Laminin subunit alpha-3 | -0.18 | 0.02 | 0.25 |
| 202527_s_at | 4089 | SMAD4 | Mothers against decapentaplegic homolog 4 | -0.17 | 0.02 | 0.27 |
| 223278_at | 2706 | GJB2 | Gap junction beta-2 protein | -0.17 | 0.02 | 0.27 |
| 1555812_a_at | 397 | ARHGDIB | Rho GDP-dissociation inhibitor 2 | -0.17 | 0.03 | 0.30 |
| 202193_at | 3985 | LIMK2 | LIM domain kinase 2 | -0.17 | 0.03 | 0.30 |
| 206343_s_at | 3084 | NRG1 | Pro-neuregulin-1, membrane-bound isoform | -0.16 | 0.03 | 0.31 |
| 226535_at | 3694 | ITGB6 | Integrin beta-6 | -0.16 | 0.03 | 0.31 |
| 210367_s_at | 9536 | PTGES | Prostaglandin E synthase | -0.15 | 0.03 | 0.33 |
| 33322_i_at | 2810 | SFN | 14-3-3 protein sigma | -0.15 | 0.03 | 0.30 |
| 204165_at | 8936 | WASF1 | Wiskott-Aldrich syndrome protein family member 1 | -0.15 | 0.04 | 0.37 |
| 204446_s_at | 240 | ALOX5 | Arachidonate 5-lipoxygenase | -0.15 | 0.04 | 0.37 |
| 32137_at | 3714 | JAG2 | Protein jagged-2 | -0.15 | 0.05 | 0.38 |
| 217109_at | 4585 | MUC4 | Mucin-4 | -0.15 | 0.05 | 0.38 |
| 201015_s_at | 3728 | JUP | Junction plakoglobin | -0.14 | 0.05 | 0.38 |
| 212543_at | 202 | AIM1 | Absent in melanoma 1 protein | -0.14 | 0.05 | 0.38 |
| 203304_at | 25805 | BAMBI | BMP and activin membrane-bound inhibitor homolog | 0.30 | 0.00 | 0.01 |
| 202609_at | 2059 | EPS8 | Epidermal growth factor receptor kinase substrate 8 | 0.25 | 0.00 | 0.05 |
| 201105_at | 3956 | LGALS1 | Galectin-1 | 0.21 | 0.00 | 0.14 |
| 218678_at | 10763 | NES | Nestin | 0.19 | 0.01 | 0.18 |
| 216598_s_at | 6347 | CCL2 | C-C motif chemokine 2 | 0.19 | 0.01 | 0.22 |
| 200633_at | 7314 | UBB | Polyubiquitin-B | 0.19 | 0.01 | 0.22 |
| 221911_at | 2115 | ETV1 | ETS translocation variant 1 | 0.18 | 0.01 | 0.22 |
| 211518_s_at | 652 | BMP4 | Bone morphogenetic protein 4 | 0.18 | 0.01 | 0.22 |
| 204115_at | 2791 | GNG11 | Guanine nucleotide-binding protein G(I)/G(S)/G(O) subunit gamma-11 | 0.18 | 0.01 | 0.22 |
| 212298_at | 8829 | NRP1 | Neuropilin-1 | 0.18 | 0.02 | 0.27 |
| 221731_x_at | 1462 | VCAN | Versican core protein | 0.18 | 0.02 | 0.28 |
| 212154_at | 6383 | SDC2 | Syndecan-2 | 0.17 | 0.03 | 0.31 |
| 201508_at | 3487 | IGFBP4 | Insulin-like growth factor-binding protein 4 | 0.17 | 0.03 | 0.31 |
| 204584_at | 3897 | L1CAM | Neural cell adhesion molecule L1 | 0.17 | 0.02 | 0.28 |
| 201426_s_at | 7431 | VIM | Vimentin | 0.16 | 0.03 | 0.32 |
| 225564_at | 221178 | SPATA13 | Spermatogenesis-associated protein 13 | 0.16 | 0.04 | 0.34 |
| 202403_s_at | 1278 | COL1A2 | Collagen alpha-2(I) chain | 0.15 | 0.03 | 0.32 |
| 221558_s_at | 51176 | LEF1 | Lymphoid enhancer-binding factor 1 | 0.15 | 0.03 | 0.33 |
| 224917_at | 406991 | MIR21 | Mature microRNA-21-5p | 0.15 | 0.05 | 0.37 |
| 209118_s_at | 7846 | TUBA1A | Tubulin alpha-1A chain | 0.14 | 0.05 | 0.38 |
| 203989_x_at | 2149 | F2R | Proteinase-activated receptor 1 | 0.14 | 0.05 | 0.39 |
