# Supplementary material for: Development of a Drug-Response Modeling Framework to Identify Cell Line Derived Translational Biomarkers That Can Predict Treatment Outcome to Erlotinib or Sorafenib
Source: PLoS One. 2015 Jun 24;10(6):e0130700. doi: 10.1371/journal.pone.0130700 (PMC4480971; doi:10.1371/journal.pone.0130700)
Supplement: S2 Table — A raw p-value of each gene was calculated during feature selection step based on the fitted mean and standard deviation from the permutation data (see Methods in the main text section). Adjusted p-value was calculated using Benjamini-Hochberg control of false discovery rate. (DOCX) [file pone.0130700.s010.docx]

Table S2: the 113 gene Sorafenib signature genes.

A raw p-value of each gene was calculated during feature selection step based on the fitted mean and standard deviation from the permutation data (see Methods in the main text section). Adjusted p-value was calculated using Benjamini-Hochberg control of false discovery rate.

| **ProbeSet ID** | **EntrezGene ID** | **Gene Name** | **Protein name** | **Correlation coefficient** | **p-value** | **p-value adjusted** |
| --- | --- | --- | --- | --- | --- | --- |
| 203131_at | 5156 | PDGFRA | Platelet-derived growth factor receptor alpha | -0.42 | 0.00 | 0.00 |
| 219213_at | 58494 | JAM2 | Junctional adhesion molecule B | -0.38 | 0.00 | 0.00 |
| 211535_s_at | 2260 | FGFR1 | Fibroblast growth factor receptor 1 | -0.32 | 0.00 | 0.01 |
| 209815_at | 5727 | PTCH1 | Protein patched homolog 1 | -0.28 | 0.00 | 0.04 |
| 210755_at | 3082 | HGF | Hepatocyte growth factor | -0.26 | 0.00 | 0.05 |
| 202283_at | 5176 | SERPINF1 | Pigment epithelium-derived factor | -0.26 | 0.00 | 0.05 |
| 203666_at | 6387 | CXCL12 | Stromal cell-derived factor 1 | -0.25 | 0.00 | 0.06 |
| 203130_s_at | 3800 | KIF5C | Kinesin heavy chain isoform 5C | -0.23 | 0.01 | 0.10 |
| 202527_s_at | 4089 | SMAD4 | Mothers against decapentaplegic homolog 4 | -0.22 | 0.01 | 0.09 |
| 215076_s_at | 1281 | COL3A1 | Collagen alpha-1(III) chain | -0.19 | 0.03 | 0.15 |
| 204412_s_at | 4744 | NEFH | Neurofilament heavy polypeptide | -0.19 | 0.03 | 0.17 |
| 231951_at | 2775 | GNAO1 | Guanine nucleotide-binding protein G(o) subunit alpha | -0.18 | 0.04 | 0.20 |
| 201474_s_at | 3675 | ITGA3 | Integrin alpha-3 | 0.44 | 0.00 | 0.00 |
| 225330_at | 3480 | IGF1R | Insulin-like growth factor 1 receptor | 0.44 | 0.00 | 0.00 |
| 225927_at | 4214 | MAP3K1 | Mitogen-activated protein kinase kinase kinase 1 | 0.39 | 0.00 | 0.00 |
| 209140_x_at | 3106 | HLA-B | HLA class I histocompatibility antigen, B | 0.37 | 0.00 | 0.01 |
| 203510_at | 4233 | MET | Hepatocyte growth factor receptor | 0.34 | 0.00 | 0.01 |
| 228121_at | 7042 | TGFB2 | Transforming growth factor beta-2 | 0.34 | 0.00 | 0.01 |
| 227396_at | 5795 | PTPRJ | Receptor-type tyrosine-protein phosphatase eta | 0.33 | 0.00 | 0.01 |
| 203324_s_at | 858 | CAV2 | Caveolin-2 | 0.32 | 0.00 | 0.01 |
| 202668_at | 1948 | EFNB2 | Ephrin-B2 | 0.32 | 0.00 | 0.02 |
| 212242_at | 7277 | TUBA4A | Tubulin alpha-4A chain | 0.32 | 0.00 | 0.02 |
| 212097_at | 857 | CAV1 | Caveolin-1 | 0.31 | 0.00 | 0.03 |
| 209270_at | 3914 | LAMB3 | Laminin subunit beta-3 | 0.31 | 0.00 | 0.02 |
| 202688_at | 8743 | TNFSF10 | Tumor necrosis factor ligand superfamily member 10 | 0.31 | 0.00 | 0.02 |
| 211651_s_at | 3912 | LAMB1 | Laminin subunit beta-1 | 0.31 | 0.00 | 0.02 |
| 203726_s_at | 3909 | LAMA3 | Laminin subunit alpha-3 | 0.30 | 0.00 | 0.03 |
| 201865_x_at | 2908 | NR3C1 | Glucocorticoid receptor | 0.30 | 0.00 | 0.03 |
| 211075_s_at | 961 | CD47 | Leukocyte surface antigen CD47 | 0.30 | 0.00 | 0.03 |
| 200983_x_at | 966 | CD59 | CD59 glycoprotein | 0.30 | 0.00 | 0.03 |
| 205016_at | 7039 | TGFA | Protransforming growth factor alpha | 0.30 | 0.00 | 0.03 |
| 202267_at | 3918 | LAMC2 | Laminin subunit gamma-2 | 0.29 | 0.00 | 0.03 |
| 227458_at | 29126 | CD274 | Programmed cell death 1 ligand 1 | 0.29 | 0.00 | 0.03 |
| 215313_x_at | 3105 | HLA-A | HLA class I histocompatibility antigen, A | 0.29 | 0.00 | 0.03 |
| 201109_s_at | 7057 | THBS1 | Thrombospondin-1 | 0.29 | 0.00 | 0.03 |
| 1555950_a_at | 1604 | CD55 | Complement decay-accelerating factor | 0.29 | 0.00 | 0.03 |
| 208614_s_at | 2317 | FLNB | Filamin-B | 0.29 | 0.00 | 0.03 |
| 202357_s_at | 629 | CFB | Complement factor B | 0.29 | 0.00 | 0.03 |
| 203854_at | 3426 | CFI | Complement factor I | 0.28 | 0.00 | 0.04 |
| 226535_at | 3694 | ITGB6 | Integrin beta-6 | 0.28 | 0.00 | 0.03 |
| 223000_s_at | 50848 | F11R | Junctional adhesion molecule A | 0.28 | 0.00 | 0.03 |
| 212195_at | 3572 | IL6ST | Interleukin-6 receptor subunit beta | 0.28 | 0.00 | 0.04 |
| 210095_s_at | 3486 | IGFBP3 | Insulin-like growth factor-binding protein 3 | 0.28 | 0.00 | 0.04 |
| 212464_s_at | 2335 | FN1 | Fibronectin | 0.27 | 0.00 | 0.04 |
| 203837_at | 4217 | MAP3K5 | Mitogen-activated protein kinase kinase kinase 5 | 0.27 | 0.00 | 0.04 |
| 209619_at | 972 | CD74 | HLA class II histocompatibility antigen gamma chain | 0.27 | 0.00 | 0.05 |
| 205767_at | 2069 | EREG | Proepiregulin | 0.27 | 0.00 | 0.05 |
| 208812_x_at | 3107 | HLA-C | HLA class I histocompatibility antigen, Cw | 0.27 | 0.00 | 0.05 |
| 232151_at | 346389 | MACC1 | Metastasis-associated in colon cancer protein 1 | 0.27 | 0.00 | 0.05 |
| 209016_s_at | 3855 | KRT7 | Keratin, type II cytoskeletal 7 | 0.26 | 0.00 | 0.05 |
| 209040_s_at | 5696 | PSMB8 | Proteasome subunit beta type-8 | 0.26 | 0.01 | 0.06 |
| 203973_s_at | 1052 | CEBPD | CCAAT/enhancer-binding protein delta | 0.26 | 0.00 | 0.05 |
| 202286_s_at | 4070 | TACSTD2 | Tumor-associated calcium signal transducer 2 | 0.26 | 0.00 | 0.05 |
| 232231_at | 860 | RUNX2 | Runt-related transcription factor 2 | 0.26 | 0.00 | 0.05 |
| 201650_at | 3880 | KRT19 | Keratin, type I cytoskeletal 19 | 0.26 | 0.01 | 0.06 |
| 208949_s_at | 3958 | LGALS3 | Galectin-3 | 0.26 | 0.00 | 0.05 |
| 218368_s_at | 51330 | TNFRSF12A | Tumor necrosis factor receptor superfamily member 12A | 0.26 | 0.00 | 0.05 |
| 217767_at | 718 | C3 | Complement C3 | 0.26 | 0.01 | 0.06 |
| 211756_at | 5744 | PTHLH | Parathyroid hormone-related protein | 0.25 | 0.01 | 0.06 |
| 204990_s_at | 3691 | ITGB4 | Integrin beta-4 | 0.25 | 0.01 | 0.06 |
| 203440_at | 1000 | CDH2 | Cadherin-2 | 0.25 | 0.00 | 0.06 |
| 211964_at | 1284 | COL4A2 | Collagen alpha-2(IV) chain | 0.25 | 0.01 | 0.06 |
| 212236_x_at | 3872 | KRT17 | Keratin, type I cytoskeletal 17 | 0.25 | 0.01 | 0.06 |
| 206336_at | 6372 | CXCL6 | C-X-C motif chemokine 6 | 0.25 | 0.01 | 0.06 |
| 202071_at | 6385 | SDC4 | Syndecan-4 | 0.25 | 0.01 | 0.06 |
| 209185_s_at | 8660 | IRS2 | Insulin receptor substrate 2 | 0.25 | 0.01 | 0.06 |
| 205239_at | 374 | AREG | Amphiregulin | 0.25 | 0.01 | 0.06 |
| 227314_at | 3673 | ITGA2 | Integrin alpha-2 | 0.24 | 0.01 | 0.06 |
| 203407_at | 5493 | PPL | Periplakin | 0.24 | 0.01 | 0.07 |
| 201189_s_at | 3710 | ITPR3 | Inositol 1,4,5-trisphosphate receptor type 3 | 0.24 | 0.01 | 0.08 |
| 204584_at | 3897 | L1CAM | Neural cell adhesion molecule L1 | 0.24 | 0.01 | 0.07 |
| 205207_at | 3569 | IL6 | Interleukin-6 | 0.24 | 0.01 | 0.07 |
| 204259_at | 4316 | MMP7 | Matrilysin | 0.24 | 0.01 | 0.08 |
| 203987_at | 8323 | FZD6 | Frizzled-6 | 0.24 | 0.01 | 0.08 |
| 208510_s_at | 5468 | PPARG | Peroxisome proliferator-activated receptor gamma | 0.24 | 0.01 | 0.08 |
| 204470_at | 2919 | CXCL1 | Growth-regulated alpha protein | 0.24 | 0.01 | 0.07 |
| 201860_s_at | 5327 | PLAT | Tissue-type plasminogen activator | 0.24 | 0.01 | 0.09 |
| 202023_at | 1942 | EFNA1 | Ephrin-A1 | 0.23 | 0.01 | 0.10 |
| 201131_s_at | 999 | CDH1 | Cadherin-1 | 0.23 | 0.01 | 0.10 |
| 222549_at | 9076 | CLDN1 | Claudin-1 | 0.23 | 0.01 | 0.10 |
| 201428_at | 1364 | CLDN4 | Claudin-4 | 0.23 | 0.01 | 0.10 |
| 216379_x_at | 100133941 | CD24 | Signal transducer CD24 | 0.23 | 0.01 | 0.08 |
| 204748_at | 5743 | PTGS2 | Prostaglandin G/H synthase 2 | 0.22 | 0.01 | 0.10 |
| 205490_x_at | 2707 | GJB3 | Gap junction beta-3 protein | 0.22 | 0.02 | 0.11 |
| 206295_at | 3606 | IL18 | Interleukin-18 | 0.22 | 0.01 | 0.10 |
| 228188_at | 2355 | FOSL2 | Fos-related antigen 2 | 0.22 | 0.02 | 0.11 |
| 209212_s_at | 688 | KLF5 | Krueppel-like factor 5 | 0.22 | 0.01 | 0.10 |
| 204115_at | 2791 | GNG11 | Guanine nucleotide-binding protein G(I)/G(S)/G(O) subunit gamma-11 | 0.21 | 0.02 | 0.12 |
| 210986_s_at | 7168 | TPM1 | Tropomyosin alpha-1 chain | 0.21 | 0.02 | 0.12 |
| 210764_s_at | 3491 | CYR61 | Protein CYR61 | 0.21 | 0.02 | 0.13 |
| 202826_at | 6692 | SPINT1 | Kunitz-type protease inhibitor 1 | 0.21 | 0.02 | 0.11 |
| 203821_at | 1839 | HBEGF | Heparin-binding EGF-like growth factor | 0.21 | 0.02 | 0.12 |
| 225973_at | 6891 | TAP2 | Antigen peptide transporter 2 | 0.21 | 0.03 | 0.15 |
| 201015_s_at | 3728 | JUP | Junction plakoglobin | 0.21 | 0.03 | 0.15 |
| 211980_at | 1282 | COL4A1 | Collagen alpha-1(IV) chain | 0.20 | 0.02 | 0.12 |
| 201983_s_at | 1956 | EGFR | Epidermal growth factor receptor | 0.20 | 0.02 | 0.11 |
| 217728_at | 6277 | S100A6 | Protein S100-A6 | 0.20 | 0.03 | 0.15 |
| 209774_x_at | 2920 | CXCL2 | C-X-C motif chemokine 2 | 0.20 | 0.03 | 0.15 |
| 209894_at | 3953 | LEPR | Leptin receptor | 0.20 | 0.02 | 0.12 |
| 203411_s_at | 4000 | LMNA | Prelamin-A/C | 0.20 | 0.03 | 0.16 |
| 223278_at | 2706 | GJB2 | Gap junction beta-2 protein | 0.19 | 0.03 | 0.17 |
| 210118_s_at | 3552 | IL1A | Interleukin-1 alpha | 0.19 | 0.04 | 0.18 |
| 210367_s_at | 9536 | PTGES | Prostaglandin E synthase | 0.19 | 0.05 | 0.20 |
| 204279_at | 5698 | PSMB9 | Proteasome subunit beta type-9 | 0.19 | 0.04 | 0.20 |
| 201596_x_at | 3875 | KRT18 | Keratin, type I cytoskeletal 18 | 0.18 | 0.04 | 0.18 |
| 207717_s_at | 5318 | PKP2 | Plakophilin-2 | 0.18 | 0.04 | 0.18 |
| 219395_at | 80004 | ESRP2 | Epithelial splicing regulatory protein 2 | 0.18 | 0.04 | 0.19 |
| 205479_s_at | 5328 | PLAU | Urokinase-type plasminogen activator | 0.18 | 0.05 | 0.21 |
| 203395_s_at | 3280 | HES1 | Transcription factor HES-1 | 0.18 | 0.04 | 0.20 |
| 224833_at | 2113 | ETS1 | Protein C-ets-1 | 0.18 | 0.04 | 0.18 |
| 217109_at | 4585 | MUC4 | Mucin-4 | 0.18 | 0.05 | 0.21 |
| 201655_s_at | 3339 | HSPG2 | Basement membrane-specific heparan sulfate proteoglycan core protein | 0.18 | 0.05 | 0.21 |
| 205302_at | 3484 | IGFBP1 | Insulin-like growth factor-binding protein 1 | 0.18 | 0.04 | 0.20 |
