# Supplementary material for: Development of a Drug-Response Modeling Framework to Identify Cell Line Derived Translational Biomarkers That Can Predict Treatment Outcome to Erlotinib or Sorafenib
Source: PLoS One. 2015 Jun 24;10(6):e0130700. doi: 10.1371/journal.pone.0130700 (PMC4480971; doi:10.1371/journal.pone.0130700)
Supplement: S3 Table — Pearson’s correlation coefficient was calculated for a vector of log2(IC50) and a vector of genetic events. Positive value of correlation for a given gene was interpreted as correlation with resistance. Negative value can was interpreted as an association with sensitivity. Correlation P-values were calculated by a permutation test with 1000 shuffles. Fisher’s exact test was used for association analysis between resistance/sensitivity classes and genetic events. “R” stands for resistance, “S” stands for sensitivity. (DOCX) [file pone.0130700.s011.docx]

Table S3. Association analysis of genetic events and drug response

Pearson’s correlation coefficient was calculated for a vector of log2(IC50) and a vector of genetic events. Positive value of correlation for a given gene was interpreted as correlation with resistance. Negative value can was interpreted as an association with sensitivity. Correlation P-values were calculated by a permutation test with 1000 shuffles. Fisher’s exact test was used for association analysis between resistance/sensitivity classes and genetic events.

“R” stands for resistance, “S” stands for sensitivity.

| Gene | Erlotinib | | | | | | | | | | | | | | | | Sorafenib | | | | | | | | | | | | | | | |
| --- | --- | --- | --- | --- | --- | --- | --- | --- | --- | --- | --- | --- | --- | --- | --- | --- | --- | --- | --- | --- | --- | --- | --- | --- | --- | --- | --- | --- | --- | --- | --- | --- |
|  | Correlation analysis ("R" resistance-specific, "S" sensitivity-specific) | | | | | | |  | Association analysis, Fisher's exact test ("R" resistance-specific, "S" sensitivity-specific) | | | | | | | | Correlation analysis ("R" resistance-specific, "S" sensitivity-specific) | | | | | | | | Association analysis, Fisher's exact test ("R" resistance-specific, "S" sensitivity-specific) | | | | | | | |
|  | Any genetic events | p-value | Deletions | p-value | Mutations | p-value | Amplifications | p-value | Any genetic events | p-value | Deletions | p-value | Mutations | p-value | Amplifications | p-value | Any genetic events | p-value | Deletions | p-value | Mutations | p-value | Amplifications | p-value | Any genetic events | p-value | Deletions | p-value | Mutations | p-value | Amplifications | p-value |
| KRAS | R | 0.007 |  |  | R | 0.007 |  |  |  |  |  |  |  |  |  |  |  |  |  |  |  |  |  |  |  |  |  |  |  |  |  |  |
| PTEN | R | 0.019 |  |  |  |  |  |  |  |  |  |  |  |  |  |  |  |  |  |  |  |  |  |  | R | 0.030 |  |  |  |  |  |  |
| EGFR | S | 0.012 |  |  | S | 0.013 |  |  | S | 0.037 |  |  | S | 0.023 |  |  |  |  |  |  |  |  |  |  | R | 0.028 |  |  |  |  |  |  |
| RB1 |  |  |  |  |  |  | S | 0.029 |  |  |  |  |  |  |  |  |  |  |  |  |  |  |  |  | R | 0.032 |  |  |  |  |  |  |
| BRCA2 | S | 0.008 |  |  |  |  | S | 0.028 | S | 0.020 |  |  |  |  |  |  |  |  |  |  |  |  |  |  |  |  |  |  |  |  |  |  |
| FLT3 | S | 0.022 |  |  |  |  | S | 0.019 |  |  |  |  |  |  |  |  |  |  |  |  |  |  |  |  |  |  |  |  |  |  |  |  |
| CTNNB1 | S | 0.05 |  |  |  |  |  |  |  |  |  |  |  |  |  |  | S | 0.035 |  |  |  |  |  |  |  |  |  |  |  |  |  |  |
| PIK3CA |  |  |  |  |  |  | S | 0.03 |  |  |  |  |  |  |  |  |  |  |  |  |  |  |  |  |  |  |  |  |  |  |  |  |
| SMAD4 |  |  |  |  |  |  |  |  |  |  |  |  |  |  |  |  | R | 0.016 |  |  |  |  |  |  |  |  |  |  |  |  |  |  |
| MET |  |  |  |  |  |  |  |  |  |  |  |  |  |  |  |  | R | 0.039 |  |  |  |  | R | 0.036 |  |  |  |  |  |  |  |  |
| SMO |  |  |  |  |  |  |  |  |  |  |  |  |  |  |  |  | R | 0.04 |  |  |  |  | R | 0.046 |  |  |  |  |  |  |  |  |
| NF2 |  |  |  |  |  |  |  |  |  |  |  |  |  |  |  |  |  |  |  |  |  |  |  |  |  |  |  |  | R | 0.049 |  |  |
| SMARCA4 |  |  |  |  |  |  |  |  |  |  |  |  |  |  |  |  |  |  |  |  |  |  |  |  | S | 0.024 |  |  | S | 0.049 |  |  |
