# Supplementary material for: Development of a Drug-Response Modeling Framework to Identify Cell Line Derived Translational Biomarkers That Can Predict Treatment Outcome to Erlotinib or Sorafenib
Source: PLoS One. 2015 Jun 24;10(6):e0130700. doi: 10.1371/journal.pone.0130700 (PMC4480971; doi:10.1371/journal.pone.0130700)
Supplement: S1 Text — Description and discussion of reconstructed causal models omitted from main manuscript and shown in S3 and S4 Fig and Fig 2 in the main manuscript section. (DOCX) [file pone.0130700.s012.docx]

**Supplementary text 1.** Description of causal models reconstructed from Erlotinib and Sorafenib predictive models

**Functional analysis of the Erlotinib and Sorafenib predictive models and insights into drug mechanisms of action**

In order to understand how the signature genes are related to the biology of drug response, we reconstructed a causal network using the genes from the predictive models and high fidelity human protein interactions and molecular canonical pathways from MetaBase^TM^ and Pathway Maps ontology (Thomson Reuters) (see Methods section in the main section). The resulted molecular networks (S3 and S4 Figures) visualize interrelationships between signature genes* and represent the hypotheses on sensitivity and resistance. The hypothesis could be experimentally validated and employed in drug discovery and therapy development research. Functional overview of the reconstructed networks with literature references are presented below.

**Further the term “sensitivity-specific gene” refers to genes with expression negatively correlated with IC50; the term “resistance-specific gene” refers to genes with expression positively correlated with IC50. Therefore these phenotype-specific genes show either higher expression in cells with the phenotype, or show lower expression in cells with the opposite phenotype.*

**Causal model reconstructed from Erlotinib predictive model (S3 Figure)**

The network reconstructed from Erlotinib predictive model illustrates cross-talk between Erlotinib signaling and parallel/cross-talking pathways.

Erlotinib binds ErbB-EGFR and so inhibits the downstream molecular network*. E.g.,* EGFR family members induce numerous cancer pathways, including the ones transduced via ERK1/2 and PI3K ([1](#_ENREF_1)), as shown in the network. ErbB-EGFR is regulated by sensitivity-specific ligand Neuregulin 1 ([2-5](#_ENREF_2)), included in Erlotinib prediction model. In addition, correlation and association analyses of genetic data reveals correlation between EGFR mutations with sensitive phenotype among OncoPanel cell lines. Taking into account expression and genetic data, one can suggest disturbance of EGFR family signaling at genetic and expression level in sensitive cells. Administration of Erlotinib is supposed to inhibit EGFR-related signaling, which manifests as sensitive response to treatment and supported by clinical observaions. Specifically, EGFR mutations predispose better response to Erlotinib in BATTLE study ([6](#_ENREF_6)), and in other studies related to progression free survival ([7](#_ENREF_7)). Interestingly, EGFR amplification also provides survival benefits for Erlotinib treated patients ([8](#_ENREF_8)).

Besides ErbB-EGFR regulators, Erlotinib prediction model includes other genes related to sensitive phenotype, including enzymes involved in prostaglandins biosynthesis (enzymes COX-1, PGES), regulators of NOTCH signaling (Jagged2, DLL1), extracellular matrix proteins, receptors of adhesion and intracellular cytoskeleton proteins (proteins of Laminin 5 group, ITGB6, P-Cadherin, Keratins *etc*.). EGFR-regulated molecular network includes all these genes as nodes on the canonical pathways enriched with the genes from Erlotinib prediction model. Specifically, the network shows regulation of EGFR signaling by prostaglandins and adhesion receptors, whilst NOTCH can be downstream of ErbB signaling (see also references on the subject ([9-14](#_ENREF_9))). Altogether, the network demonstrates the complexity of sensitivity-related signaling pathways directly or indirectly modulated by EGFR family and, therefore, targeted by Erlotinib in sensitive cells.

Although the reconstructed molecular network illustrates probable functional interrelations between the genes of Erlotinib predictive model, alternative regulatory circuits can be regulated by sensitivity-specific genes in specific cancer types or in peculiar cell lines. The mechanisms could be additionally validated in focused cancer type or data set-specific experiments.

Erlotinib prediction model includes a number of resistance-specific ligands, receptors and transcription factors (including Syndecan-2, Neuropilin-1, PAR1, CCL2, BMP4, Lef-1, microRNA 21) integrated in molecular pathways **cross-talking** with, **parallel** to or **regulated** by ErbB-EGFR molecular network. High expression of the genes in resistant cells could activate the corresponding signaling pathways. These molecular pathways cannot be directly inhibited by Erlotinib and so decrease effectiveness of Erlotinib, providing cells with resistant phenotype.

Similar interpretation could be applied to other genes with genetic events significantly related to resistant phenotype and included in the reconstructed pathways. Here we focus on a particularly interesting example, K-RAS. K-RAS mutations in OncoPanel cell lines correlates with resistant phenotype (Table S3). We suggest that disturbance of the downstream pathway, e.g. related to downstream activation of ERK1/2, could endow cells with higher insensitivity to EGFR inhibitors, which act upstream of K-RAS. Clinical data on significance of K-RAS mutations for Erlotinib response is ambiguous. On the one hand, BATTLE and other studies ([6](#_ENREF_6), [15](#_ENREF_15)) failed to demonstrate significant relation of K-RAS mutations occurrence with drug response. On the other hand, some clinical reports highlight relation of wild type K-RAS to certain survival of progression-free survival benefits from Erlotinib therap. Such inconsistency suggests high complexity of factors which alone or in combination could influence drug response. The conditions which could predispose negative influence of mutated K-RAS and its downstream signaling for Erlotinib response need more research and validation.

To summarize, the reconstructed networks illustrate the disturbance of ErbB-EGFR signaling in cancer cells as the underlying mechanism of Erlotinib sensitivity, whilst activation of cross-talking, parallel and downstream pathways decrease effectiveness of Erlotinib, thus underlying resistance. The revealed interactions are in line with modern understanding of resistance to EGFR inhibitors, which is generally considered to be caused by accumulation of mutations in genes related to regulation of EGFR signaling, overactivation of cross talking pathways, VEGF and IGF-R signaling, and deregulation of PI3K signaling due to different reasons (reviewed in ([16](#_ENREF_16), [17](#_ENREF_17))).

**Causal model reconstructed from Sorafenib predictive model (S4 Figure)**

The network reconstructed from Sorafenib predictive model genes illustrates diversity and distinctive regulation of highly interconnected intracellular network in sensitive and resistant cells.

Sorafenib is a multikinase inhibitor with a wide range of inhibitory activity (with different potency) against serine/threonine protein kinases Rafs and a number of receptor tyrosine kinases ([18-20](#_ENREF_18)). Multitargeting ability of Sorafenib suggests multiple mechanisms of drug action and drug resistance, which could be distinct in individual cases.

Sorafenib predictive model includes sensitivity-specific PDGF-R-alpha and FGFR1, receptors with tyrosine kinase activity targeted by Sorafenib. The kinase receptors induce multiple downstream signaling pathways ([21](#_ENREF_21)) and those enriched with signature genes are shown in the reconstructed network. PDGF-Rs and FGFR1 also trigger downstream activation of ERK1/2 via RAFs kinases. This pathway is also targeted by Sorafenib (RAF inhibition). Interestingly, there are no sensitivity-specific mark-ups in downstream signaling pathways. Therefore, similarly to Erlotinib case, sensitivity-specific cells are characterized by higher expression of drug targets.

Sorafenib prediction model includes resistance-specific tyrosine kinase receptors EGFR, IGF-1 receptor, HGF receptor, which are not or weekly (case of HGF receptor) inhibited by Sorafenib ([20](#_ENREF_20)), as opposed to sensitivity-specific PDGF-R-alpha and FGFR1. Moreover, the prediction model includes resistance-specific EGFR ligands - Amphiregulin, Epiregulin, TGF-alpha, HB-EGF, suggesting possibility of autocrine regulation of EGFR pathway in resistance cells. In addition, genetic events in EGFR gene (not shown in the network) in cell lines of OncoPanel panel correlate with resistant response providing additional supportive evidence on significance of EGFR pathway disturbance for Sorafenib response. To summarize, Sorafenib resistant cells tend to have disturbance of EGFR signaling at expression and genetic levels. This observation is supported by clinical data. Specifically, mutations and polysomy of EGFR indicated worse response to Sorafenib in BATTLE study ([6](#_ENREF_6)). Besides the described resistance-specific tyrosine kinase receptors, Sorafenib prediction model also includes other genes encoding interleukins, chemokines and adhesion receptors. We suppose that higher expression of the tyrosine kinase receptors or other receptors/lingands insensitive (or with lower sensitivity) to Sorafenib inhibition as well as genetic changes in the corresponding genes could trigger downstream cancer signaling pathways and therefore insensitive to Sorafenib treatment.

The resistance-specific receptors and ligands, not inhibited by Sorafenib can induce molecular pathways **cross-talking** with or **parallel** to drug targets signaling. In the reconstructed network we show pathways enriched with genes from Sorafenib prediction model or affected by genes with drug response significant genetic events. The expression and genetic changes at the level of pathway regulators could cause changes of activity of the whole pathways and their contribution to cancer progression resulting in insensitivity to Sorafenib. Here we provide an example of such pathways.

One of suggested resistance-specific signaling pathway is mediated through resistance-specific MEKK1, cross-talking with RAF-induced pathways targeted by Sorafenib. In this case, MEKK1 functions positively contribute to cancer progression, the resistance-specific cross-talk is expected to reduce effectiveness of Sorafenib therapy. MEKK1 is known to be indispensable for survival and anti-apoptosis in pancreatic ([22](#_ENREF_22)) and leukemia ([23](#_ENREF_23)) cancer cells. In addition to survival, MEKK1 could be involved in other pathways significant for pathogenesis, *e.g.* it positively regulates invasion and migration in pancreatic cancer cells ([24](#_ENREF_24)). However, hypothetical positive contribution of MEKK1 signaling to cancer progression and survival could be specific to a biological context, as generally MEKK1 is considered as a regulator of pro-apoptotic molecular pathways (see as an example references ([25-28](#_ENREF_25))), which is opposed to the suggested scheme of resistance mechanisms. We address to this function of MEKK1 in paragraphs below.

Interestingly, Sorafenib prediction signature includes a number of genes with expression positively correlated with IC50 (resistance-specific), which shown as inductors of apoptosis in enriched signaling pathways or/and reference as such in GO knowledge database: Lamin A/C, ASK1, MEKK1, FN14, Apo-2L. Expression of these genes may convey pre- or apoptotic physiological state of “resistant” cells. This can mean that the phenotype of such cells is not “truly” resistant, as they were undergoing apoptosis at the end of the experiment. Alternatively, the expression of the genes should be analyzed along with genetic status of key regulators of apoptosis to test whether the expression profile marks cells undergoing apoptosis, or those which are characterized with disrupted apoptosis regulation despite the high level of the discussed apoptosis regulators.

In total, the reconstructed network suggests a variety of pathways interesting for further focused experimental investigation in relation to the drug response. Significance of some pathways finds support in literature, while others are harder to interpret and demand additional analysis to ensure the relevance of the pathway to drug response.

To summarize, the reconstructed network illustrates signaling pathways through PDGF-Rs and FGFR1 sensitive to Sorafenib, and signaling pathways triggered by ligands and receptors not targeted by Sorafenib and therefore resistant to it.

**References**

1. Hynes NE, MacDonald G. ErbB receptors and signaling pathways in cancer. Current opinion in cell biology. 2009;21(2):177-84. doi: 10.1016/j.ceb.2008.12.010. PubMed PMID: 19208461.

2. Britsch S, Li L, Kirchhoff S, Theuring F, Brinkmann V, Birchmeier C, et al. The ErbB2 and ErbB3 receptors and their ligand, neuregulin-1, are essential for development of the sympathetic nervous system. Gene Dev. 1998;12(12):1825-36. doi: DOI 10.1101/gad.12.12.1825. PubMed PMID: WOS:000074505300009.

3. Riese DJ, Vanraaij TM, Plowman GD, Andrews GC, Stern DF. The Cellular-Response to Neuregulins Is Governed by Complex Interactions of the Erbb Receptor Family. Mol Cell Biol. 1995;15(10):5770-6. PubMed PMID: WOS:A1995RV77200062.

4. Stove C, Bracke M. Roles for neuregulins in human cancer. Clinical & experimental metastasis. 2004;21(8):665-84. PubMed PMID: 16035612.

5. Holbro T, Beerli RR, Maurer F, Koziczak M, Barbas CF, Hynes NE. The ErbB2/ErbB3 heterodimer functions as an oncogenic unit: ErbB2 requires ErbB3 to drive breast tumor cell proliferation. P Natl Acad Sci USA. 2003;100(15):8933-8. doi: DOI 10.1073/pnas.1537685100. PubMed PMID: WOS:000184371000062.

6. Kim ES, Herbst RS, Wistuba II, Lee JJ, Blumenschein GR, Tsao A, et al. The BATTLE Trial: Personalizing Therapy for Lung Cancer. Cancer Discov. 2011;1(1):44-53. doi: Doi 10.1158/2159-8274.Cd-10-0010. PubMed PMID: WOS:000295780300021.

7. Brugger W, Triller N, Blasinska-Morawiec M, Curescu S, Sakalauskas R, Manikhas GM, et al. Prospective molecular marker analyses of EGFR and KRAS from a randomized, placebo-controlled study of erlotinib maintenance therapy in advanced non-small-cell lung cancer. Journal of clinical oncology : official journal of the American Society of Clinical Oncology. 2011;29(31):4113-20. doi: 10.1200/JCO.2010.31.8162. PubMed PMID: 21969500.

8. Zhu CQ, Santos GD, Ding K, Sakurada A, Cutz JC, Liu N, et al. Role of KRAS and EGFR as biomarkers of response to erlotinib in National Cancer Institute of Canada Clinical Trials Group study BR.21. Journal of Clinical Oncology. 2008;26(26):4268-75. doi: Doi 10.1200/Jco.2007.14.8924. PubMed PMID: WOS:000259350400008.

9. Falcioni R, Antonini A, Nistico P, DiStefano S, Crescenzi M, Natali PG, et al. alpha 6 beta 4 and alpha 6 beta 1 integrins associate with ErbB-2 in human carcinoma cell lines. Exp Cell Res. 1997;236(1):76-85. doi: DOI 10.1006/excr.1997.3695. PubMed PMID: WOS:A1997YC72400009.

10. Fedor-Chaiken M, Hein PW, Stewart JC, Brackenbury R, Kinch MS. E-cadherin binding modulates EGF receptor activation. Cell Commun Adhes. 2003;10(2):105-18. doi: Doi 10.1080/15419060390261058. PubMed PMID: WOS:000187367400004.

11. Moro L, Dolce L, Cabodi S, Bergatto E, Erba EB, Smeriglio M, et al. Integrin-induced epidermal growth factor (EGF) receptor activation requires c-Src and p130Cas and leads to phosphorylation of specific EGF receptor tyrosines. J Biol Chem. 2002;277(11):9405-14. doi: DOI 10.1074/jbc.M109101200. PubMed PMID: WOS:000174400600090.

12. Ding YB, Shi RH, Tong JD, Li XY, Zhang GX, Xiao WM, et al. PGE2 up-regulates vascular endothelial growth factor expression in MKN28 gastric cancer cells via epidermal growth factor receptor signaling system. Experimental oncology. 2005;27(2):108-13. PubMed PMID: 15995627.

13. Lindsay J, Jiao X, Sakamaki T, Casimiro MC, Shirley LA, Tran TH, et al. ErbB2 induces Notch1 activity and function in breast cancer cells. Clinical and translational science. 2008;1(2):107-15. doi: 10.1111/j.1752-8062.2008.00041.x. PubMed PMID: 20443831; PubMed Central PMCID: PMC3590841.

14. Pradeep CR, Kostler WJ, Lauriola M, Granit RZ, Zhang F, Jacob-Hirsch J, et al. Modeling ductal carcinoma in situ: a HER2-Notch3 collaboration enables luminal filling. Oncogene. 2012;31(7):907-17. doi: 10.1038/onc.2011.279. PubMed PMID: 21743488; PubMed Central PMCID: PMC3193899.

15. Langer CJ. Roles of EGFR and KRAS Mutations in the Treatment Of Patients With Non-Small-Cell Lung Cancer. P T. 2011;36(5):263-79. Epub 2011/07/26. PubMed PMID: 21785539; PubMed Central PMCID: PMC3138369.

16. Bar J, Onn A. Overcoming molecular mechanisms of resistance to first-generation epidermal growth factor receptor tyrosine kinase inhibitors. Clinical lung cancer. 2012;13(4):267-79. doi: 10.1016/j.cllc.2011.09.001. PubMed PMID: 22154113.

17. Wheeler DL, Dunn EF, Harari PM. Understanding resistance to EGFR inhibitors-impact on future treatment strategies. Nature reviews Clinical oncology. 2010;7(9):493-507. doi: 10.1038/nrclinonc.2010.97. PubMed PMID: 20551942; PubMed Central PMCID: PMC2929287.

18. Adnane L, Trail PA, Taylor I, Wilhelm SM. Sorafenib (BAY 43-9006, Nexavar), a dual-action inhibitor that targets RAF/MEK/ERK pathway in tumor cells and tyrosine kinases VEGFR/PDGFR in tumor vasculature. Methods in enzymology. 2006;407:597-612. doi: 10.1016/S0076-6879(05)07047-3. PubMed PMID: 16757355.

19. Wilhelm SM, Carter C, Tang L, Wilkie D, McNabola A, Rong H, et al. BAY 43-9006 exhibits broad spectrum oral antitumor activity and targets the RAF/MEK/ERK pathway and receptor tyrosine kinases involved in tumor progression and angiogenesis. Cancer research. 2004;64(19):7099-109. doi: 10.1158/0008-5472.CAN-04-1443. PubMed PMID: 15466206.

20. Karaman MW, Herrgard S, Treiber DK, Gallant P, Atteridge CE, Campbell BT, et al. A quantitative analysis of kinase inhibitor selectivity. Nature biotechnology. 2008;26(1):127-32. doi: 10.1038/nbt1358. PubMed PMID: 18183025.

21. Lemmon MA, Schlessinger J. Cell signaling by receptor tyrosine kinases. Cell. 2010;141(7):1117-34. doi: 10.1016/j.cell.2010.06.011. PubMed PMID: 20602996; PubMed Central PMCID: PMC2914105.

22. Hirano T, Shino Y, Saito T, Komoda F, Okutomi Y, Takeda A, et al. Dominant negative MEKK1 inhibits survival of pancreatic cancer cells. Oncogene. 2002;21(38):5923-8. doi: 10.1038/sj.onc.1205643. PubMed PMID: 12185592.

23. Nawata R, Yujiri T, Nakamura Y, Ariyoshi K, Takahashi T, Sato Y, et al. MEK kinase 1 mediates the antiapoptotic effect of the Bcr-Abl oncogene through NF-kappaB activation. Oncogene. 2003;22(49):7774-80. doi: 10.1038/sj.onc.1206901. PubMed PMID: 14586403.

24. Su F, Li H, Yan C, Jia B, Zhang Y, Chen X. Depleting MEKK1 expression inhibits the ability of invasion and migration of human pancreatic cancer cells. Journal of cancer research and clinical oncology. 2009;135(12):1655-63. doi: 10.1007/s00432-009-0612-6. PubMed PMID: 19513748.

25. Fuchs SY, Adler V, Pincus MR, Ronai Z. MEKK1/JNK signaling stabilizes and activates p53. Proc Natl Acad Sci U S A. 1998;95(18):10541-6. PubMed PMID: 9724739; PubMed Central PMCID: PMC27930.

26. Abreu-Martin MT, Chari A, Palladino AA, Craft NA, Sawyers CL. Mitogen-activated protein kinase kinase kinase 1 activates androgen receptor-dependent transcription and apoptosis in prostate cancer. Mol Cell Biol. 1999;19(7):5143-54. PubMed PMID: 10373563; PubMed Central PMCID: PMC84357.

27. Boldt S, Weidle UH, Kolch W. The kinase domain of MEKK1 induces apoptosis by dysregulation of MAP kinase pathways. Exp Cell Res. 2003;283(1):80-90. PubMed PMID: 12565821.

28. Komoda F, Shino Y, Hirano T, Okutomi Y, Okamoto H, Hayashi Y, et al. MEKK1 induces c-Jun complexes that act as negative regulators for cell survival and proliferation of HCC cells. International journal of oncology. 2002;21(3):553-9. PubMed PMID: 12168099.
