# Supplementary material for: Development of a Drug-Response Modeling Framework to Identify Cell Line Derived Translational Biomarkers That Can Predict Treatment Outcome to Erlotinib or Sorafenib
Source: PLoS One. 2015 Jun 24;10(6):e0130700. doi: 10.1371/journal.pone.0130700 (PMC4480971; doi:10.1371/journal.pone.0130700)
Supplement: S2 Text — Additional results and methods omitted from the main manuscript. We provide results of association analysis between genetic events and drug response supporting major observations in the manuscript. (DOCX) [file pone.0130700.s013.docx]

**Association analysis between genetic events and drug response**

Correlation/association analysis of genetic data from Sanger Institute database (see Cancer Cell Line Project at <http://www.sanger.ac.uk/genetics/CGP/CellLines/>) collected data on various genetic alterations in cancer cell lines. We used the available data for 62 genes on amplifications (copy numbers) for 127 cell lines and the data on mutations for 130 cell lines of OncoPanel.

For each of 62 genes Pearson’s correlation coefficient was calculated for a vector of log2(IC50) and a vector of genetic events. Correlation P-values were calculated by a permutation test (1000 shuffles). Correlation p-values were used to detect the genes that can be involved in the sensitivity/resistance mechanisms. P-value threshold 0.05 was chosen to make an assumption that the genetic events for a given gene are associated with compounds sensitivity or resistance. Positive value of correlation for a given gene was interpreted as correlation with resistance. Negative value can was interpreted as an association with sensitivity. For each of 62 genes association analysis between resistance/sensitivity classes and genetic events was performed using Fisher’s exact test. The following null hypothesis was tested: cell lines with a particular type of genetic event in a given gene are randomly distributed across two groups of cell lines – sensitive and resistant.
